# Amyloid‐Like Protein Aggregation Toward Pesticide Reduction

**DOI:** 10.1002/advs.202105106

**Published:** 2022-03-08

**Authors:** Hao Su, Yongchun Liu, Yingtao Gao, Chengyu Fu, Chen Li, Rongrong Qin, Lei Liang, Peng Yang

**Affiliations:** ^1^ Key Laboratory of Applied Surface and Colloid Chemistry Ministry of Education School of Chemistry and Chemical Engineering Shaanxi Normal University Xi'an 710119 China; ^2^ School of Chemistry and Chemical Engineering Henan Institute of Science and Technology Eastern HuaLan Avenue Xinxiang Henan 453003 China

**Keywords:** amyloid, droplet deposition, pesticide reduction, protein membrane, superhydrophobic surface

## Abstract

Pesticide overuse is a major global problem and the cause of this problem is noticeable pesticide loss from undesired bouncing of sprayed pesticide droplets and rain erosion. This further becomes a primary source of soil and groundwater pollution. Herein, the authors report a method that can enhance pesticide droplet deposition and adhesion on superhydrophobic plant leave surfaces by amyloid‐like aggregation of bovine serum albumin (BSA). Through the reduction of the disulfide bond of BSA by tris(2‐carboxyethyl) phosphine hydrochloride (TCEP), the amyloid‐like phase transition of BSA is triggered that rapidly affords abundant phase‐transitioned BSA (PTB) oligomers to facilitate the invasion of the PTB droplet into the nanostructures on a leaf surface. Such easy penetration is further followed by a robust amyloid‐mediated interfacial adhesion of PTB on leaf surface. As a result, after mixing with pesticides, the PTB system exhibits a remarkable pesticide adhesion capacity that is more than 10 times higher than conventional fixation of commercial pesticides. The practical farmland experiments show that the use of PTB aggregation could reduce the use of pesticides by 70–90% while ensuring yield. This work demonstrates that current pesticide dosage in actual agriculture production may be largely reduced by utilizing eco‐friendly amyloid‐like protein aggregation.

## Graphical abstract

Ecosystem pollution resulted from agricultural pesticide overuse poses an increasing threat to human and animal health. Herein, the authors present a groundbreaking strategy for decreasing consumption of pesticides that uses amyloid‐like protein aggregation to enhance droplet deposition and strengthen the adhesion force between plant leaves and pesticides simultaneously. In this way, normal usage of pesticides is reduced effectively by ≈70%–90%.

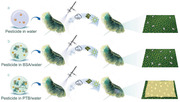

## 1 Introduction

Contemporary agriculture faces one of the greatest challenges of the 21st century: reducing agricultural damage to the environment while ensuring food production to feed a growing population.^[^ ^1^ ^]^ Reducing pesticide use is one of the key drivers to meet this challenge.^[^ ^2^ ^]^ Notable levels of pesticides have been found in ecosystems such as shallow groundwater and drinking water in all agricultural regions around the world.^[^ ^3^ ^]^ Such situations occur because more than 50% of agrochemicals are lost due to undesired rebounding and splashing behaviors when the chemicals impact hydrophobic/superhydrophobic plant leaves having a low‐surface‐energy wax layer and a topography with roughness at the micro/nanoscale.^[^ ^4^, ^5^, ^6^, ^7^, ^8^, ^9^ ^]^ Even though some pesticide droplets could attach onto plant leaves with improved suspension formula or technology, most of pesticide would be further easily washed away under rain erosion due to the lack of a strong interaction between pesticides and hydrophobic plant leave surfaces.^[^ ^28^ ^]^ As a result, it causes not only overuse of pesticides but also serious environmental pollution after the pesticides enter into the environment,^[^ ^10^, ^11^, ^12^, ^13^ ^]^ having harmful effects on human health^[^ ^14^, ^15^ ^]^ as well as endangering species and ecosystems.^[^ ^16^, ^17^ ^]^ In this regard, an efficient and environmentally friendly method to enhance pesticide liquids deposition and adhesion on plant leave surfaces is highly desirable but remains a great challenge.

The enhancement of pesticide drop retention is co‐contributed from two aspects, including 1) the droplet deposition and 2) pesticide adhesion to resist rainfall erosion. For example, the use of synthetic polymers,^[^ ^9^, ^18^, ^22^ ^]^ surfactants,^[^ ^19^, ^20^, ^21^, ^22^, ^69^, ^70^, ^71^ ^]^ charged droplets,^[^ ^23^, ^24^, ^25^, ^26^ ^]^ TiO_2_ hydrophilic nanoparticles,^[^ ^72^ ^]^ Folate/Zinc supramolecular hydrogels,^[^ ^73^ ^]^ or nanofibers assembled from natural glycyrrhizic acid^[^ ^74^ ^]^ could enhance 1) on the hydrophobic or superhydrophobic surfaces, while the use of nanocarriers such as high energy electron beam (HEEB)‐modified attapulgite aggregates,^[^ ^27^, ^28^ ^]^ plant ash,^[^ ^75^ ^]^ polydopamine nanoparticles,^[^ ^29^ ^]^ or phosphorylated zein (P‐zein)^[^ ^30^ ^]^ enhances 2). Although trimeric surfactant^[^ ^31^ ^]^ and castor oil‐based cationic/anionic polyurethane dispersions (WPU)^[^ ^32^ ^]^ were used to increase droplet deposition while immobilizing pesticides have been reported, they are not ideal systems for pesticides retention due to their complex synthesis processes and low fixation efficiency. Moreover, the extensive use of synthetic surfactants and polymers further induces secondary pollution in ecosystems due to their potential physiological toxicity and poor degradation properties.^[^ ^33^, ^34^ ^]^ Alternatively, natural surfactants such as proteins and essential oils have been added into pesticide formula to enhance the dispersion of hydrophobic pesticide and wetting of pesticide droplets on leaf surface. However, due to the lack of strong interactions with leaf surface, these natural additives could not strengthen effectively pesticide adhesion on leaf surface to resist water fluid erosion. Based on the above considerations, an efficient strategy relying on natural safe material to simultaneously enhance both of droplet deposition and pesticide adhesion is highly demanding and remains a major challenge to resolving the issues of pesticide overuse.

**Scheme 1:**
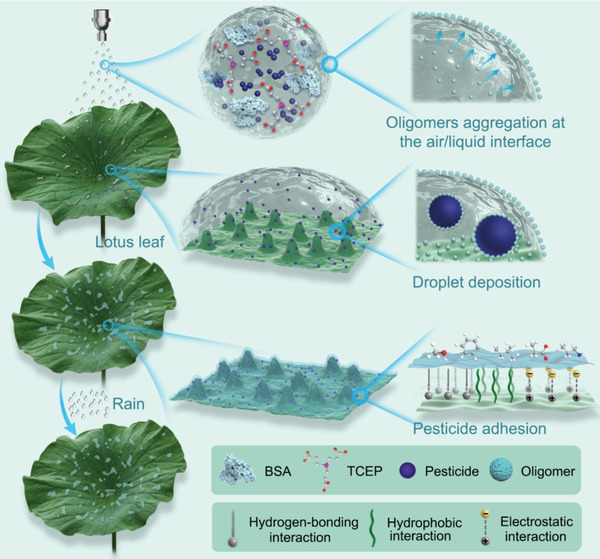
The process of pesticide droplet deposition and adhesion to lotus leaf through PTB aggregations.

Herein, we propose an approach to enhance pesticide retention by simultaneously enhancing droplet deposition and pesticide adhesion based on amyloid‐like protein aggregation (**Scheme** **1**). In this system, bovine serum albumin (BSA) can undergo rapid amyloid‐like aggregation after the reduction of its disulfide bond by the reducing agent tris(2‐carboxyethyl) phosphine (TCEP). The resulting phase‐transitioned BSA (PTB) aggregation enhances droplet deposition by enriching the PTB oligomers at the pesticide droplet interface and then assembles at liquid/solid interface to fix the pesticides on plant surfaces by forming a nanofilm on plant leave surfaces. The mechanism behind this phenomenon and corresponding results are distinctive from the previous work on the PTB‐based antifouling coating,^[^ ^37^ ^]^ highlighting the finding that the PTB droplets could break through the air cushion of a superhydrophobic surface and thus invade into the nanostructures on plant leave. This concept is effectively applied to most types of pesticides currently used worldwide and significantly enhances the pesticide fixation amount on a wide range of common plants to a level that is outstanding compared with conventional commercial pesticide formula (more than 10 times higher than commercial pesticide fixation). In addition, the biocompatibility of the proteinaceous PTB nanofilm is helpful for maintaining a low environmental risk. More importantly, a practical farmland experiment is further performed to demonstrate that the development of the present concept favors a 70–90% decrease in the amount of pesticide used in crop planting. The present work demonstrates that the amyloid‐like aggregation of unfolded proteins has much more powerful capability than conventional surfactant formula to enhance pesticide efficacy and reduce its overuse. This result thus further exploits a pathway that the manipulation of protein conformation change to form protein aggregates is a transformative strategy to enhance matter retention on a (super)hydrophobic surface and reduce corresponding matter waste during deposition process. As described below, the logical order of this work is as follows: 1) droplet impacting to leaf surface, 2) droplet adhering to leaf surface after impacting, 3) the stability evaluation of droplet after adhering to leaf surface, 4) the practical efficacy to reduce pesticide overuse in laboratory and real farmland by this strategy.

## 2 Results and Discussion

### 2.1 Phase‐Transitioned BSA (PTB) Solution and Its Sticky Behavior on Lotus Leaves

**Figure 1:**
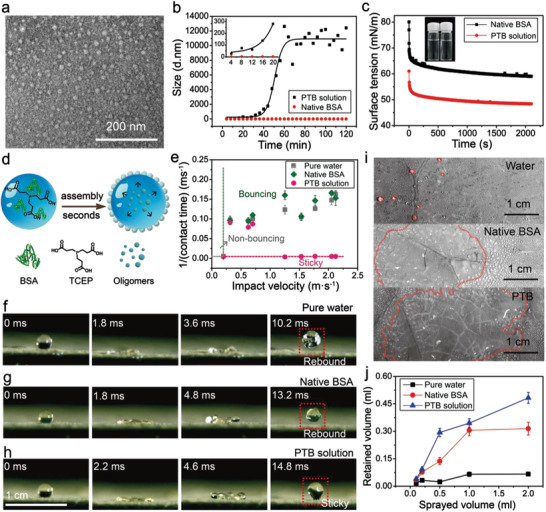
The bouncing and pinning behavior of droplets on lotus leaves. a) TEM image of the as‐prepared PTB solution incubated for 4 min. b) Time‐dependent size evolution of colloids in the as‐prepared PTB solution with native BSA solution as a control. Inset, the size evolution of colloids in the as‐prepared PTB solution in ≈20 min. c) Dynamic surface tensions of native BSA solution and the as‐prepared PTB solution. Optical photograph in the inset showing the native BSA solution and the as‐prepared PTB solution (from left to right). d) Schematic illustration for oligomer formation in the as‐prepared PTB droplet. e) Inverse of the impact contact time plotted versus the impact velocity. The green dotted line means critical impact velocity delimiting the bouncing‐nonbouncing transition. The pink dotted line means critical impact velocity delimiting the bouncing‐sticky transition. f–h) Impact process of a water droplet f), a native BSA droplet g) and an as‐prepared PTB solution droplet h) on the superhydrophobic leaf surface. i) High‐speed camera screenshots of the sprays of pure water, native BSA solution and the as‐prepared PTB solution on lotus leaves. The red curves indicate the formation of droplet deposition on the lotus leaves. j) Retained volume of liquid on a lotus leaf surface after spraying pure water, native BSA, or the as‐prepared PTB solution.

The PTB solution was freshly prepared by mixing BSA (10 mg mL^−1^) and TCEP (50 × 10^−3^ m, pH 4.5) solution. Upon mixing, the native *α*‐helix structure of BSA could rapidly convert to *β*‐sheet stacking after reduction of the disulfide bond of BSA by TCEP, leading to the amyloid‐like aggregation of unfolded BSA chains.^[^ ^35^, ^36^, ^37^ ^]^ The resultant PTB oligomer particles grew rapidly from 30 nm at 4 min to 300 nm at 20 min and gradually attained a plateau at >10 µm at 60 min, as reflected by transmission electron microscopy (TEM) and dynamic light scattering (DLS) measurements (**Figure** **1**a,b). The surface tension of native BSA solution decreased to 53 mN m^−1^ from 65 mN m^−1^ in 30 s after adding TCEP (Figure 1c), because nanoscale oligomer particles rapidly aggregated at the air/water interface to effectively reduce the interfacial free energy.^[^ ^38^ ^]^ This result is consistent with the findings of the phase‐transitioned lysozyme in previous work as characterized via Constrained Drop Surfactometry (CDS), in which it was found that the phase‐transitioned lysozyme oligomer nanoparticles aggregated at the droplet interface to decrease surface tension (Figure 1d).^[^ ^36^ ^]^ Such interfacial enrichment of protein oligomer nanoparticles would further enhance droplet wetting on leaf surface. As a result, when the aqueous droplets of pure water, native BSA solution and as‐prepared PTB solution were sprayed onto the lotus leaf surface, a strong droplet pinning on the lotus leaf was observed for the PTB solution. The statistical analysis quantified that the water droplet, BSA droplet and PTB droplet did not bounce anymore when the impact speed being less than 0.2 m s^−1^, while all droplets bounced off from the lotus leaf surface when the impact speed is between 0.2 and 1.25 m s^−1^ (Figure 1e). This is because the initial kinetic energy of the droplet is insufficient to overcome the surface energy stored when the droplet expands and retracts on such a superhydrophobic surface.^[^ ^39^, ^40^, ^41^ ^]^ Then, when the impact speed is higher than 1.25 m s^−1^, it was clearly observed that the water droplet and native BSA droplet still bounced off from the lotus leaf surface (Figure 1f,g, and Movies S1 and S2, Supporting Information), while the as‐prepared PTB solution droplets presented strong pinning on the lotus leaf (Figure 1h; and Movie S3, Supporting Information). For the PTB dispersion droplet, a reduction of surface tension in the droplet through the enrichment of oligomer particles at the droplet interface could offset the upward capillary force to break through the air cushion and facilitate the invasion of the PTB droplet into the nanostructures on the lotus leaf.^[^ ^20^, ^41^, ^42^, ^43^, ^44^, ^45^ ^]^

It should be noted that the sticky effect of the PTB droplet could not be achieved by simply using surface‐active proteins or peptides (e.g., casein).^[^ ^37^ ^]^ In the present case, the key for the PTB droplet breaking through the air cushion inside micro/nano structures on superhydrophobic surface contains double aspects: 1) the rapid decrease of surface tension of the PTB droplet caused by the phase transition reaction of BSA and 2) amyloid‐mediated strong interfacial adhesion of the phase‐transitioned proteins. In this regard, the protein phase transition is highly required in the present strategy. According to previous work,^[^ ^36^, ^47^ ^]^ a protein phase transition could occur only when globular proteins meet all the following conditions: 1) the existence of high fibrillation propensity (HFP) segment, 2) abundant *α*‐helix structures, and 3) intramolecular S—S bonds to lock the *α*‐helix. With this respect, common surface‐active molecules do not certainly fulfill all of these requirements, and it is hard for common surface‐active molecules without protein phase transition to stably adhere onto hydrophobic leaf surface during the short impact time.^[^ ^20^, ^37^, ^77^ ^]^ As shown in Figure 1i, the differentiated bounding behaviors of droplets then resulted in a lower splashing extent for the as‐prepared PTB solution than native BSA solution, forming large‐area spreading of the PTB droplets on lotus leaf, while pure water droplets presented intensive splashing from the leaf surface to form tiny liquid droplets (Movies S4–S6, Supporting Information). On a lotus leaf surface, at most 0.65 mL of water and 0.3 mL of native BSA solution was retained respectively as the spraying volume increased. In contrast, when the PTB solution was sprayed on the lotus leaf surface, the retained volume could continuously increase, and such retained volume only stopped to increase when the sample area is eventually not large enough to hold more liquid. The volume of the as‐prepared PTB solution retained on the lotus leaf surface was >12 times and 2 times higher than those of pure water and native BSA solution after 0.5 mL were sprayed, respectively (Figure 1j). In this regard, the as‐prepared PTB solution exhibited powerful capability to enhance droplet deposition on the superhydrophobic surface.

### 2.2 The Formation of PTB Nanofilm on a Leaf Surface

**Figure 2:**
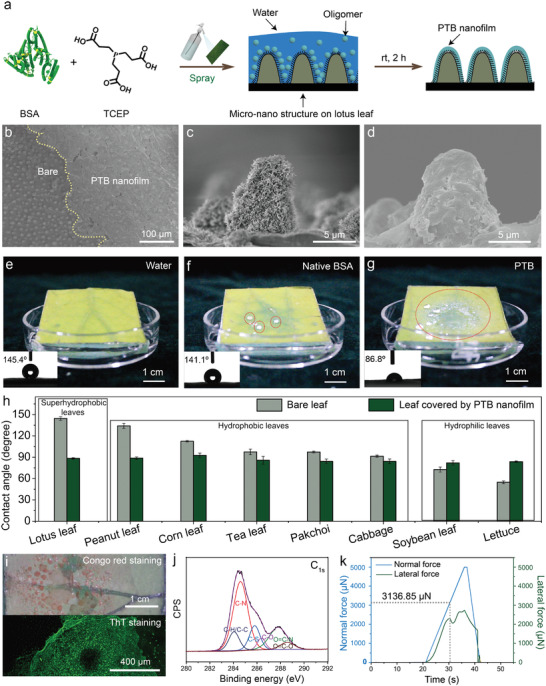
The formation of a PTB nanofilm on a lotus leaf. a) Schematic illustration for PTB nanofilm formation on the lotus leaf. b) SEM images of lotus leaf covered by the PTB nanofilm. For clarity, the image was taken near the borderline between the PTB coating area and bare lotus leaf surface (as indicated by a curve). c) The SEM image of the cross‐section of the bare lotus leaf micropillar. d) The SEM image of the cross‐section of the lotus leaf micropillar covered by the PTB nanofilm. e–g) Screenshots of the lotus leave surfaces after spraying pure water e), native BSA solution f), and the as‐prepared PTB solution g), incubating under ambient conditions for 120 min and rinsing with 6 mL of water. The droplet deposition area after rinsing in the groups of native BSA solution and as‐prepared PTB solution is indicated by a dotted circle. Insets are the corresponding WCA images on lotus leaf surfaces in different groups after spraying, incubating and rinsing with water. h) The water contact angles on a variety of bare plant leaf surfaces and leaf surfaces modified by the PTB nanofilm. i) The Congo red staining and ThT staining of the PTB nanofilm on lotus leaves. j) High‐resolution XPS C_1s_ spectra on the PTB nanofilm. k) The adhesion evaluation of the PTB nanofilm on bare Si substrates after spraying, incubating, and washing by copious water.

To grow a PTB nanofilm, the as‐prepared PTB mixture is sprayed on a leaf surface and incubated for a determined time at room temperature and ambient humidity (**Figure** **2**a). Easy retention of the as‐prepared PTB solution droplets during incubation created an ideal microenvironment for depositing PTB oligomer particles on a leaf surface, which in turn favored the formation of a continuous and dense PTB nanofilm (Figure 2b). It can be seen from SEM cross‐section images, the micro–nano structures on lotus leaf were wrapped by PTB membrane because PTB solution can enter into the micro–nano structures by breaking through the air cushion in these micro–nano structures of leaf surface (Figure 2c,d), while such action was not apparent in the groups of pure water or native BSA (Figure S1, Supporting Information). The coating thickness on a model substrate (Si wafer) was measured as ≈1–90 nm depending on the pH and concentration of the TCEP solution as well as the incubation time and was largely thickened from 46 nm to 1.7 µm as the BSA concentration increased from 2 to 60 mg mL^−1^ (Figure S2, Supporting Information). The water contact angle (WCA) on the lotus leaf surface in the PTB group decreased significantly to 86.8° from the initial 141.8°, which was in sharp contrast to the pure water and native BSA solution groups presenting no obvious WCA changes on the lotus leaf surfaces (Figure 2e–g; and Movies S7–S9, Supporting Information). The obvious hydrophilicity change occurring on the lotus leaf surface thus indicated a successful surface modification by the PTB nanofilm. Besides the lotus leaf, the PTB nanofilm was also applicable to modify other leave surfaces of plants with superhydrophobic, hydrophobic or hydrophilic properties, including cabbage, lettuce, pak choi, corn, peanuts, soybeans and tea (Figure 2h). On these PTB‐modified leave surfaces, all the WCA remained in ≈80°–90°. It should be noted that surface water contact angle is affected by a variety of factors such as surface polarity and topography structure.^[^ ^46^ ^]^ Accordingly, surface water contact angle might not be simply correlated with surface wetting capability of the PTB. The robust interfacial adhesion of the PTB layer was attributed to the existence of amyloid‐like structures inside the PTB coating.^[^ ^47^ ^]^ Thioflavin T (ThT) and Congo red staining confirmed the formation of amyloids in the nanofilm coated on the lotus leaf by presenting a green color under a confocal laser scanning microscope (CLSM) due to the specific binding of ThT to the growing *β*‐sheet structure and visible red color due to the Congo red binding with the *β*‐sheet structure (Figure 2i).^[^ ^48^, ^49^, ^50^, ^51^ ^]^ Although amyloid‐mediated interfacial adhesion mechanism is still not clear until now, it was suggested that the exposure of a variety of chemical structures by amyloid aggregation played an important role to interact with solid surface.^[^ ^52^, ^53^ ^]^ As characterized by X‐ray photoelectron spectroscopy (XPS) (Figure 2j; and Figure S3, Supporting Information), versatile hydrophilic and hydrophobic structures, typically including alphatic carbon (C—H/C—C), amines (C—N), hydroxyls (C—O), thiols (C—S), amides (O = C—N), and carboxyl groups (O═C—O) were simultaneously distributed on the PTB nanofilm, which may form multiplex binding with plant surfaces to enhance the interfacial adhesion through hydrogen bonding, electrostatic, and hydrophobic interactions.^[^ ^47^, ^54^ ^]^ Based on these multiple interactions, the as‐prepared PTB showed much higher adsorption affinity toward lotus leaf surface than did the native BSA (20 times enhancement), as reflected by the bicinchoninic acid (BCA) protein assay (Figure S4, Supporting Information). The interfacial adhesion strength of the PTB nanofilm was then quantified around 3.1 mN by a nanoscratch tester (Figure 2k), which was comparable with those from synthetic and natural coatings.^[^ ^55^, ^56^ ^]^

**Figure 3:**
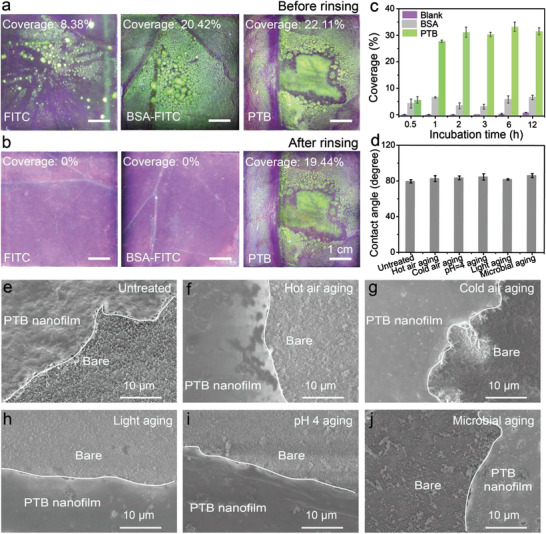
Water erosion resistance and weather resistance of PTB nanofilm. a,b) Fluorescent images of lotus surfaces (5 × 5 cm^2^) with spraying 500 µL of FITC solution, native BSA‐FITC solution or as‐prepared PTB‐FITC solution and incubation at ambient condition before a) and after b) submerging in plenty of water and shaking for 10 min. c) Surface coverage of the lotus leaf surface by the PTB solution after incubating for different time. d) The water contact angle of the PTB nanofilm on the nano‐SiO_2_ superhydrophobic surface before (untreated) and after treating with different aging conditions. e–j) The weatherability test of the PTB nanofilm. SEM image of the nano‐SiO_2_ superhydrophobic coating covered by the PTB nanofilm with different treatment. For clarity, the imaging is taken near the borderline between the PTB nanofilm area and bare nano‐SiO_2_ superhydrophobic surface (as indicated by the white curve).

The surface coverage of the PTB after spraying was further evaluated by using BSA conjugated with fluorescein isothiocyanate (BSA‐FITC) as the starting material. Through imaging under an ultraviolet light, the coverage was calculated as coverage = *S*’/*S*, where *S*’ is the area of the surface covered by the as‐sprayed liquid or PTB nanofilm and *S* is the total area of the entire surface (Figure S5, Supporting Information). On lotus leaves (5 × 5 cm^2^), the coverage did not exceed 8.38% after 0.5 mL pure water (with FITC added) was sprayed, and almost no water was left after the water rinse. Although the native BSA‐FITC aqueous solution showed corresponding coverage of 20.42% after spraying, this value rapidly decreased to 0% after water rinsing. In contrast, the coverage of the as‐prepared PTB‐FITC solution was 22.11% after 0.5 mL of the liquid was sprayed and only showed a slight decrease to 19.44% after the sufficient water rinse (**Figure** **3**a,b). The above experiment thus proved that the PTB nanofilm showed excellent adhesion performance on a surface compared to FITC solution and native BSA‐FITC solution at the same conditions. The surface coverage of PTB could then be controlled by reaction parameters including the pH and the concentration of the TCEP solution as well as the incubation time on the leaf surface (Figures S6–S9, Supporting Information). A pH close to the isoelectric point of native BSA (pI 4.7) to largely attenuate the colloidal surface charge, and a relatively high TCEP concentration and long incubation time could further enhance the surface coverage. The results then showed that the PTB coverage tended to stabilize at about 30% after incubating for 1 h (2 mg mL^−1^ BSA and 50 × 10^−3^ m TCEP (pH = 4.5)), while the coverage in blank group was nearly 0% and the coverage for native BSA group fluctuated around 5% at most (Figure 3c). For such a modification on the nano‐SiO_2_‐based synthetic superhydrophobic surface, the corresponding morphology of the PTB nanofilm remained unchanged under a series of aging conditions,^[^ ^57^ ^]^ such as high temperature (80 °C, 30 d), low temperature (−24 °C, 30 d), simulated natural light exposure (20 000 LX, 30 d), simulated acid rainfall (pH 4, 30 d), and microbial environments (37 °C, 90 d) (Figure 3d–j), as well as treatments with various organic solvents (Figure S10, Supporting Information).

### 2.3 Fixation of Pesticides by the PTB Nanofilm

**Figure 4:**
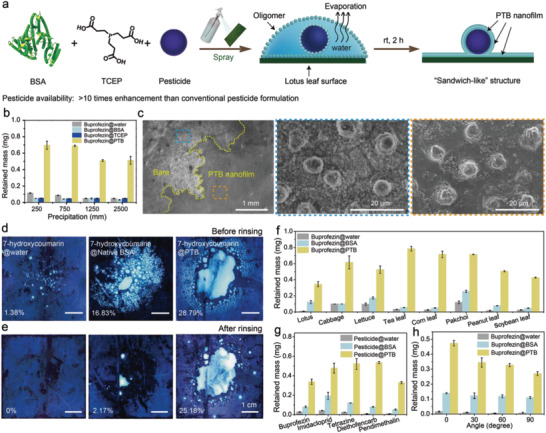
Fixation of pesticides on plant leaves. a) Schematic of pesticide fixation on lotus leaves by the PTB. b) Retained mass of buprofezin in different groups on the lotus leaves after scouring. c) SEM image of pesticides fixed on lotus leaves by the PTB (left). For clarity, the image is taken near the borderline between the PTB coating area and bare lotus leaf surface (as indicated by a yellow dotted curve). The high magnification SEM image of bare lotus leaf (middle). The high magnification SEM image of buprofezin fixed by the PTB nanofilm (right). d,e) The covering photographs of the lotus leaf with spraying (from left to right row) suspensions of 7‐hydroxycoumarin in different groups (water, native BSA solution, or PTB solution) on the lotus leaf before d) and after e) abundant water rinsing. f) Retained mass of buprofezin (95%) on common plant leaves. g) Retained mass of common pesticides fixed on lotus leaves. h) Retained mass of buprofezin (95%) on a lotus leaf a at incidental angles of 0°, 30°, 60°, and 90°.

The noticeably enhanced surface coverage of the PTB nanofilm compared with that of pure water or native protein solution after water rinsing encourages us to further use this strategy for the enhancement of pesticide retention on plant leaves, which is a major challenge in agriculture. For this purpose, a commonly‐used commercial formulated pesticide, buprofezin (50%, suspension concentrate (SC)) (Table S1, Supporting Information), was simply added to the as‐prepared phase transition solution of BSA to prepare a PTB‐modified pesticide suspension for use in a spray. After spraying such a mixture or control samples (a mixture of pure water and formulated pesticide or native BSA solution and formulated pesticide) and incubating the sprayed leaves at ambient conditions for a given time (**Figure** **4**a), the leaves were rinsed with copious water (to mimic a rain wash) and subjected to a series of characterizations. Similar to the phase transition process of BSA without the addition of pesticide, the fluorescence of the phase transition solution of BSA with buprofezin added was enhanced within 90 min, affording the PTB nanofilm on the lotus leaf surface as clearly characterized by the ThT and Congo red staining (Figure S11, Supporting Information). The results confirmed that pesticides did not affect largely the phase transition process of proteins. The experiment was conducted in lab with a scour force at 9.6 N through a self‐made device (Figure S12, Supporting Information). The scour force applied in this experiment is nearly 190 times higher than the impact force of a raindrop.^[^ ^58^ ^]^ The lotus leaves after spraying the pesticides were then repetitively scoured for 1 time, 3 times, 5 times, and 10 times in this experiment and the corresponding rainfall is 250, 750, 1250, and 2500 mm, respectively. By this process, it was shown that the retained mass of Buprofezin@PTB group is 6∼11 times higher than that of the Buprofezin@water group after scouring, the native BSA and TCEP showed a poor retention ratio of buprofezin on lotus leaves (Figure 4b). The entrapment percentage of buprofezin in water, native BSA, TCEP, and PTB groups on the lotus leaf was 1.89%, 1.25%, 1.95%, and 20.5% respectively (Figure S13, Supporting Information). The corresponding SEM images after rinsing with 2500 mm precipitation at a scour force of 9.6 N consistently exhibited the enhancement on pesticide immobilization by the PTB, since there was only a little pesticide particle on the lotus leaf surface from direct spraying of the pesticide suspension in water and native BSA solution (Figure S14, Supporting Information), while many pesticide particles were clearly encased by the PTB nanofilm on the lotus leaf surface (Figure 4c). The Fourier transform infrared (FTIR) spectra then showed the stretching bands of benzene rings at 1493, 1450, and 1400 cm^−1^ assigned to buprofezin on the lotus leaf surface, indicating successful immobilization of the pesticide on the leaf surface (Figure S15, Supporting Information).^[^ ^59^ ^]^ In order to further observe the pesticides retained by the PTB nanofilm directly, 7‐hydroxycoumarin to simulate a pesticide was sprayed on the lotus leaves for fluorescent inspection under UV excitation. The resultant fluorescence area of the PTB nanofilm was much higher than that of the pure water or native BSA solution (Figure 4d,e).

Besides the effective use in a commercial formulated pesticide (Figure S16, Supporting Information), the PTB could be also utilized as an adjuvant agent to increase the fixed mass of the pesticide technical material (TC) (pure pesticide simply diluted in pure water) on the lotus leaf surface. The results showed that the amounts of buprofezin (95% in pure water) fixed on various plant leaf surfaces by the PTB nanofilm were 5–32 times higher than those fixed by pure water or native BSA solution (Figure 4f; and Figure S17, Supporting Information). The variable enhancement ratio on different types of plant leaf surfaces might be due to the differentiated surface wettability on a variety of plant leaf surfaces. This fixation method is also applicable to a variety of widely used pesticide technical material, such as imidacloprid (95%), diethofencarb (95%), acaricide clofentezine (95%), and pendimethalin (95%) (Table S2, Supporting Information). The amounts of these pesticides fixed on lotus leaf surfaces by the PTB nanofilm were 12–66 times higher than those fixed by pure water or native BSA solution (Figure 4g). The PTB nanofilm also showed excellent fixation properties on the lotus leaf at different spraying angles (Figure 4h; and Figure S18, Supporting Information). The mass retained by the PTB nanofilm then gradually decreased as the incidental angle increased, which might because the large droplets from agglomerations of small droplets more easily rolled off from the substrate at a higher incidental angle.

The thickness of the PTB nanofilm typically ranged in ≈25–125 nm under the control of incubation time from 30 to 180 min (Figure S19, Supporting Information), and most pesticides are highly hydrophobic with particle sizes in the micrometer scales (Figure S20a, Supporting Information). Based on the adhesive behaviors of the phase‐transitioned proteins on micro/nanoparticles,^[^ ^61^ ^]^ the nanoscale PTB nanofilm could also form on the buprofezin microparticle surface after buprofezin dispersed in PTB solution (Figure S20b,c, Supporting Information). There is possible hydrogen‐bonding, hydrophobic interaction, and electrostatic interaction existing between the PTB nanofilm and buprofezin particle surface. As mentioned above, when the pesticide droplet contains BSA and TCEP dropped on a lotus leaf surface, a PTB nanofilm would also form at the corresponding contacting area. Thereby these two films including the PTB on pesticide particle surface and the PTB on leaf surface serve as adhesive layer to bind the pesticide with the leaf surface. Moreover, the PTB film formed at the air/liquid interface^[^ ^35^, ^36^, ^61^ ^]^ would also collapse and cover the surfaces of pesticide particles after water evaporates (Figure S20d, Supporting Information). Therefore, the relationship between the protein membrane and pesticide may present as a “sandwich‐like” structure (Figure 4a; and Figure S20d, Supporting Information), which was also supported by SEM images with taking buprofezin (50%, SC) as the example (Figure S21, Supporting Information).

### 2.4 Pest Control Efficacy of the Pesticide and Farmland Experiment

**Figure 5:**
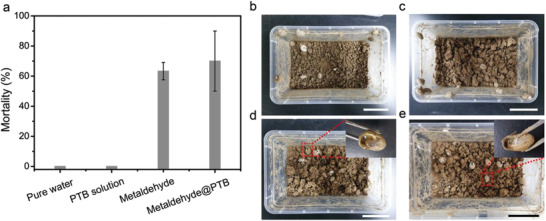
Pest control efficacy of the pesticide. a) The mortality of snails treated with different methods. b) The photograph of snails with the treatment of pure water. c) The photograph of snails with the treatment of PTB solution. d) The photograph of snails with the treatment of metaldehyde (80%, WP) suspension. The inset is the photograph of dead snail. e) The photograph of snails with the treatment of the mixture of PTB solution and metaldehyde (80%, wettable powder). The inset is the photograph of dead snail. Scale bars in b), c), d), e) are 5 cm.

Evaluation of the application value of PTB system in practical agriculture including pest control efficacy and the field efficiency of these formulations were conducted in this work. For some control studies, ^1^H nuclear magnetic resonance (NMR) and electrospray ionization mass spectrometry (ESI‐MS) characterization of metaldehyde mixed with TCEP and ^1^HNMR characterization of buprofezin immobilized by the PTB nanofilm indicated that TCEP and PTB did not have significant side effects on the molecular structure of the pesticide (Figures S22–S24, Supporting Information). Our previous work further demonstrated that the phase‐transitioned protein solution (protein and TCEP) and resultant nanofilm had excellent biocompatibility with mammalian cells and organisms.^[^ ^37^, ^57^, ^60^, ^61^ ^]^ In addition, TCEP used in the phase transition is a type of organophosphorus with low toxicity (e.g., nondetectable side effect on acetylcholinesterase activity^[^ ^62^ ^]^) and high biocompatibility,^[^ ^57^, ^61^ ^]^ which could be rapidly oxidized in air to form P‐oxide of TCEP (TCEPO)^[^ ^63^ ^]^ and further phosphate anions by phosphate solubilizing bacteria (PSB) that widely exist in soils.^[^ ^64^ ^]^ Furthermore, the PTB solution does not pose a threat to humans or nontarget organisms such as mice due to its noncytotoxicity.^[^ ^37^, ^76^ ^]^ Based on these findings, snails, typical ground pests that have a serious impact on agriculture, were used to evaluate the biological activity of metaldehyde (80%, wettable powder (WP)) in water or as‐prepared PTB solution. Metaldehyde, with the assistance of other attracting agents, would behave the killing function after snails contact with or eat metaldehyde.^[^ ^65^ ^]^ It could be seen from **Figure** **5**a that the mortality of snails is 0% with the treatment of pure water and PTB solution (Figure 5b,c), whereas the mortality of snails is around 63% with the treatment of metaldehyde suspension and the mortality of snails is around 70% with the treatment of the mixture of PTB solution and metaldehyde (Figure 5d,e). The result showed that the PTB solution is not toxic to snails and has no additional influence on the biological activity of metaldehyde.

**Figure 6:**
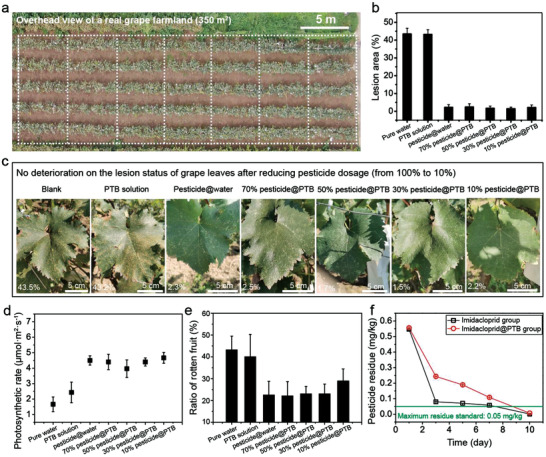
Farmland experiment. a–d) The aerial photo a), lesion area percentage b) photograph c), and photosynthetic rate d) of grape leaves in seven groups. e) The rotten fruit ratio of grapes in seven groups. f) Time‐dependent pesticide residue on corn leaves of imidacloprid in the imidacloprid suspension (Imidacloprid group) and as‐prepared PTB solution (Imidacloprid@PTB group). The green line means the maximum residue (0.05 mg kg^−1^) of imidacloprid according to the standard (GB2763‐2009). The climate and soil conditions were shown in Tables S5–S7 (Supporting Information).

The PTB aggregation is a good auxiliary agent to decrease the bound or splash of pesticide droplets, with significantly enhancing the adhesion between the pesticide and leaf surface, and in this way, the pesticide waste due to undesirable splashing during the pesticide spraying could be reasonably reduced and thus the pesticide dosage in a real farming may be decreased. Farmland experiments were then explored to verify the role of PTB aggregation in reducing pesticide overuse in actual agricultural production. As seen from the aerial photo (**Figure** **6**a), in the grape farm land experiment, seven sections were sprayed by pure water at 2.8 L (blank group), the PTB solution at 2.8 L (PTB solution group), the mixture of water and pesticide with 100% of pesticide dosage recommended by the supplier at 2.8 L (pesticide@water group), the mixture of the PTB and pesticide with 70%, 50%, 30%, and 10% of pesticide dosage recommended by the supplier at 2.8 L (70% pesticide@PTB group, 50% pesticide@PTB group, 30% pesticide@PTB group, 10% pesticide@PTB group). In this process, a combination of several types of fungicides and insecticides were used together as the pesticide to control grape diseases and pests (Table S3, Supporting Information). It takes about 10–13 min to spray a bucket of pesticide (20 L) with an artificial electric sprayer, which was then used for pesticide spraying in this experiment. In such a period, the aggregates would not clog sprinkler heads of sprayer because the size of as‐prepared PTB aggregates (Figure 1b) is similar to the size of common pesticide formulations dispersed in water (Figure S25, Supporting Information). The planting results then showed that the lesion area of grape leaves in the blank group and PTB solution group was around 43%, which rapidly decreased to 2% in the groups of pesticide@water, 70% pesticide@PTB, 50% pesticide@PTB, 30% pesticide@PTB, and 10% pesticide@PTB (Figure 6b,c). This result indicated that the pesticide@PTB group with the use of pesticide at 10% of recommended pesticide dosage achieved an efficacy similar to that of the pesticide group with the use of 100% recommended pesticide dosage. The blotch area of grape leaves in the blank and PTB solution groups would largely affect photoabsorption under natural light exposure. Since the PTB nanofilm was colorless with an optical transparency greater than 90% (Figure S26, Supporting Information), the photosynthesis rates of grape leaves in the remaining five pesticide groups remained almost at the same level, indicating little negative effect on the normal photosynthesis of plants from the PTB nanofilm. Meanwhile, without pesticide‐based insect control on grape leaves in blank and PTB solution groups, the area of diseased spots on grape leaves in blank and PTB groups were consequently so large to negatively influence photosynthesis of plants. As a result, the photosynthetic rates of leaves in blank and PTB solution groups were lower than that in other groups (Figure 6d). Further, the statistical analysis reflected that the ratio of rotten fruit in the pesticide@water group (100% pesticide dosage) remained almost at the same level with those in the groups of pesticide@PTB with the use of pesticide amount at ≈30–70% of recommended pesticide dosage (Figure 6e).

Besides the grape planting, as seen from the aerial photo (Figure S27a, Supporting Information), in the corn farmland, seven sections were sprayed by pure water at 2.2 L (blank group), the PTB solution at 2.2 L (PTB solution group), the mixture of the PTB and pesticide with 100% of pesticide dosage recommended by the supplier at 2.2 L (pesticide@water group), the mixture of the PTB and pesticide with 70%, 50%, 30%, 10% of pesticide dosage recommended by the supplier at 2.2 L (70% pesticide@PTB group, 50% pesticide@PTB group, 30% pesticide@PTB group, 10% pesticide@PTB group). A combination of several types of insecticides and fungicides were used to control corn diseases and pests (Table S4, Supporting Information). The corresponding worm‐eaten ratio of corn plants (the percentage of corn plants (both leaves and corns) that were bitten by pests to the total number of plants) in the blank group and PTB solution group was around 45%, which rapidly decreased to ≈23–28% in the groups of pesticide@water, 70% pesticide@PTB, and 50% pesticide@PTB, 30% pesticide@PTB group, and 10% pesticide@PTB group (Figure S27b, Supporting Information). This result showed that the pesticide@PTB group with the use of 10% of recommended pesticide dosage achieved an efficacy similar to that of the pesticide group with the use of 100% recommended pesticide dosage. The photosynthesis rates in the seven tested groups of corns remained almost at the same level, indicating little negative influence from the PTB nanofilm on the normal photosynthesis of plants (Figure S27c, Supporting Information) and the growth status of corns in each group was basically the same, which was supported by the plant height data collected before the corn plant height stopped growing (Figure S27d, Supporting Information).

In addition, a pore size distribution in the PTB nanofilm was measured around 70 nm (Figure S28a, Supporting Information), which thus had not high possibility to block microsized stomata on leaves.^[^ ^66^ ^]^ These nanopores in the PTB nanofilm actually originated from the voids of the PTB oligomer aggregates,^[^ ^54^, ^67^, ^68^ ^]^ and the corresponding air permeability test indicated that the porous structure of the PTB nanofilm allowed gas to be exchanged freely through nylon filter cloth and would not hinder the photosynthesis and respiration of plants (Figure S28b, Supporting Information). Meantime, these pores thus provide the effective channels to allow pesticides to enter into lipophilic channels of leaves after spraying pesticides, resulting in a normal absorption of pesticides on plant leaves.

To explore the possibility of pesticide residue caused by the PTB fixation, the concentration of imidacloprid on the corn leaves was then determined. For the fairness of the experiment, we designed the initial amount of imidacloprid in the Imidacloprid group (commercial formula) and Imidacloprid@PTB group of leaves to be the same value. After planting under a real farm condition for 10 days, the pesticide residue of imidacloprid in two groups on the corn leaves were less than the maximum residue of imidacloprid allowed by national standard (GB2763‐2009) (Figure 6f; and Table S5, Supporting Information). Accordingly, the protein membrane does not affect the natural process of pesticide digestion. Pesticide residue on plant surfaces would not be enhanced with the use of the PTB nanofilm. It has been reported that Vitamin C has the capability to insert into *β*‐sheet structure of amyloids for the disassembly, which is utilized to clean up A*β* peptide amyloid aggregates, develop smart antibacterial surface and surgical dressing.^[^ ^60^ ^]^ In addition, surfactants (ingredients of detergent) and protease also have the capacity to remove the protein membrane (Figure S29a, Supporting Information). Moreover, the surfactant may also be able to remove the proteinaceous PTB membrane from a plant leaf surface toward the removal of pesticide in daily life if necessary. The clearance ratios of pesticides from plant leave surfaces were 98.7%, 98.9%, and 26.7% by using VC solution, SDS or water as the washing solution, respectively. Meanwhile, we tested the removal ability of pesticides by some commonly‐used detergents brands in the market, showing a high clearance ratio of pesticide from plant left surface at 96.1% for Fairy (in Europe), 97.8% for KAO (in Japan) and 97.8% for myk+ (in Denmark) and 97.2% for BaiMao (in China) (Figure S29b, Supporting Information).

For another important question about cost, the retained mass of buprofezin in Buprofezin@PTB group was still 10 times higher than that of Buprofezin@water group with the use of 1 mg mL^−1^ BSA and 5 × 10^−3^ m TCEP (pH 4.5) (Figure S30a, Supporting Information). Based on a consumption of 600 L water per hectare, the single‐time spray cost per hectare for Buprofezin@PTB group is estimated at 73–77 $ (Figure S30b, Supporting Information), which was comparable with (even lower than) that of Buprofezin@water group (87–121 $). This contrast, in the combination with the above‐mentioned results, thus implied that compared with the use of commercial pesticide, the use of the PTB‐modified pesticide not only achieved almost 70–90% reduction on the dosage, but also could maintain or even lower the pesticide cost, thus holding great potential for scale‐up application to enhance the efficacy of pesticide and reduce the harm to ecosystems.

## 3 Conclusion

In this work, we propose a reliable strategy to enhance droplet deposition and adhesion on superhydrophobic surface by using a biocompatible protein aggregation system. It is found that droplet bouncing on a superhydrophobic surface could be largely suppressed by introducing the phase‐transitioned BSA (PTB) aggregate into the droplet. Such amyloid‐like protein aggregate is distinctively different from conventional surfactants, since it could simultaneously reduce surface tension of a droplet by enriching the PTB oligomer nanoparticles at a droplet interface to break through air cushion of micro/nanostructures on a surface and interact with the wetted substrate to achieve strong interfacial adhesion. Through the PTB‐assisted matter delivery, functional substances could be thus efficiently attached onto a superhydrophobic surface with largely attenuated matter loss from droplet bouncing and elution. As a practical application in pesticide immobilization on a series of widely cultivated plant leaves, the PTB‐based pesticide formulation shows an excellent pesticide fixation performance that is more than 10 times higher than conventional pesticide formulations.

It should be noted that nowadays there are typically two ways to tackle with the challenge of pesticide overuse. One is to reduce droplet splashing during pesticide spraying and strengthen the adhesion of pesticides to leaves after pesticide spraying;^[^ ^20^, ^22^, ^23^, ^74^, ^75^ ^]^ the other is to control the pesticide release into a slow long‐term course.^[^ ^29^, ^78^, ^79^ ^]^ As the present work proposes a simple, environmentally friendly and efficient method that combines the inhibition of droplet rebound with the enhancement of the interaction between pesticide and leaf surface and puts it into practice, the study to control pesticide release by such a system would be reported in the future. The specific features of the PTB being distinctive from traditional systems are as follows: 1) first, the PTB system will not cause environmental pollution on the premise of ensuring droplet deposition due to the use of biocompatible, biodegradable, and environmentally friendly protein materials, which is in sharp contrast to existing methods mostly rely on the use of synthetic polymer additives or surfactants; 2) second, this system has the advantage of simplicity and efficiency compared with the use of nanocarriers or adhesive microcapsules in conventional strategies; 3) third, also as the most different feature from existing examples being limited in laboratory exploration, we demonstrate in this work the effectiveness of this system in practical farmland (≈4 acres) to reduce the use of pesticides by 70–90% (a world‐recording value until now), realizing the technology transfer from theory to practice. The present concept is promising in a variety of surface/interface fields, such as controllable fertilization, active transdermal, cosmetics, paints, and any other process that involves droplet deposition and adhesion on (super)hydrophobic surface.

## 4 Experimental Section

### Materials and Chemicals

Bovine serum albumin (BSA), thioflavin‐T (ThT), vitamin C (VC) were purchased from Sigma‐Aldrich (St. Louis, MO). Tris (2‐carboxyethyl) phosphine hydrochloride (TCEP) was purchased from TCI (Tokyo, Japan). BSA conjugated with fluorescein isothiocyanate (BSA‐FITC) was obtained from Solarbio (Beijing, China). Congo red was purchased from Aladdin (Los Angeles, USA). Methylene chloride was purchased from Sinopharm Chemical Reagent Company (Shanghai, China). Hydrochloric acid (HCl) and sodium hydroxide (NaOH) were purchased from Sinopharm Chemical (Shanghai, China). 7‐Hydroxycoumarin (98%) was purchased from MERYER (Shanghai, China). The technical material (TC) of buprofezin (95%), imidacloprid (95%), diethofencarb (95%), pendimethalin (95%), clofentezine (95%), and the suspension concentrate (SC) of buprofezin (50%), lufenuron•chlorfenapyr (12%), thiophanate‐methyl (50%), chlorantraniliprole (20%), boscalid•iprodione (35%), thiamethoxam•lambda‐cyhalothrin (17%), methylamabamectin benzoate•mites nitrile (12%), pentazole•cytosyl ester (40%), and the water dispersible granule (WG) of imidacloprid (70%), dimethomorph•cream urea cyanide (70%), and the emulsion in water (EW) of penconazole, and the wettable powder (WP) of metaldehyde (80%) were purchased from Hailier Chemical Company (Shandong, China). Lotus leaves, tea leaves, peanut leaves, and corn seeds were purchased from Honghe Autonomous Prefecture, Yunnan Province, China. Lettuces, cabbages, pak choi were purchased from the local supermarket. The corn and grape experimental fields are located in Xi 'an, China (108° 93′ E, 34° 17′ N) and corn leaves were taken from this experimental field. Snails were purchased from Jiaxing Shangpin Food Co. Ltd. (Zhejiang, China). The nano‐SiO_2_ superhydrophobic coating was purchased from Changzhou Nanocoatings Co. Ltd (Jiangsu, China). Ultrapure water was used in all the experiments and was supplied by Milli‐Q Advantage A10 (Millipore, USA).

### Characterization

Analysis of the droplet samples on the lotus leaf surfaces and superhydrophobic substrates was recorded with IX Camera 716 high‐speed cameras. Scanning electron microscopy (SEM) observations of gold‐coated samples were made using an FE‐SEM (SU8020, Hitachi). Fourier transform infrared (FTIR) spectra (the transmission mode with the use of the KBr pellet) were obtained between 400 and 4000 cm^−1^ with a resolution of 1 cm^−1^ using an AlphaT spectrometer (Bruker). X‐ray photoelectron spectroscopy (XPS) was performed with an AXIS ULTRA from Kratos Analytical Ltd., and the binding energies were calibrated by setting the C_1s_ peak at 284.6 eV. The particle size distribution of colloids was determined by dynamic light scattering (DLS) on a Malvern Zeta sizer Nano‐ZS90. The UV–vis spectra were measured by using a UV5BIO spectrophotometer (METTLER TOLEDO). The WCA was measured on an OCA 20 (Dataphysics, Germany). The thickness of the PTB nanofilm on silicon substrates was characterized by an ALPHA‐SE ellipsometer (J.A. WOOLLAM, America). Fluorescence spectra were collected by an F‐7000 fluorescence spectrophotometer (Hitachi). The surface tension of the solution was measured by DCAT 21 (Dataphysics, Germany). A ThT‐stained PTB nanofilm on a lotus leaf was examined with a confocal laser scanning microscope (Olympus FV1200). The pesticide residue of imidacloprid was detected by gas chromatography (GC‐2010Plus, SHIMADZU). The morphology and structure of the PTB were characterized by field emission transmission electron microscopy (TEM) (Tecnai G2 F20, FEI) at 200 kV and stained with 2% w/v phosphotungstic acid aqueous solution (pH 7.0) for ≈10 min. The air permeability of the PTB nanofilm was characterized by an air permeability tester (TQD‐G1). The pore size distribution of the PTB nanofilm was measured by the BET test (ASAP 2020 HD88). The photosynthetic rates of corn and grape leaves were measured by a photosynthetic rate meter (SY‐1050, Shiya). H^1^ NMR spectra were recorded on Bruker Avance III HD 600 MHz spectrometer. HRMS was recorded on an ABI/Sciex QStar Mass Spectrometer (ESI).

### Drop Impact on the Superhydrophobic Surface

Water droplet, native BSA droplet and as‐prepared PTB solution droplet (radius 0.92 mm) were delivered by a precision needle on the lotus leaf surface with the impact velocity at 1.25 m s^−1^.

### Preparation of the PTB Solution/Nanofilm

The PTB solution was freshly prepared by mixing BSA (10 mg mL^−1^ in water) and TCEP aqueous solution (50 × 10^−3^ m TCEP in water at pH 4.5, pH adjusted by 5 m NaOH) at a volume ratio of 1:1. The mixed solution with or without the addition of pesticide was then immediately evenly sprayed on plant surfaces. After that, the solution on the surface was incubated for 30–180 min at room temperature and ambient conditions. A PTB nanofilm coating could then be formed on plant leaf surfaces by this method.

### Spray Method

The spray gun was placed 12 cm above the plant leaves to maintain a determined incidental angle (typically 30°) to the ground. The pressure from the air supply to the airbrush was kept constant throughout the whole set of experiments to maintain the same jet velocity and cone angle. The liquid was delivered to the airbrush by inputs of 500 µL liquid using a syringe. The samples (5 × 5 cm^2^) were larger than the spray area. The size of droplet sprayed by spray gun is about 500 µm.

### Coverage Determination

BSA‐FITC was used in the spray solution to image the coverage of the solution/nanofilm under ultraviolet light, and ImageJ was used to determine the area of leaves covered by the solution/nanofilm.

### Congo Red Staining and ThT Staining

Congo red staining: The lotus leaves (5 × 5 cm^2^) in the groups of water, native BSA solution and the as‐prepared PTB solution after rinse were immersed into 20 mL of 1 mg mL^−1^ Congo red solution at room temperature for 30 min. The lotus leaves were taken out and rinsed with sufficient water for 3 times, and incubated in water overnight. The stained lotus leaves were dried and imaged.^[^ ^35^ ^]^ ThT staining: The lotus leaves (5 × 5 cm^2^) in the groups of water, native BSA solution and the as‐prepared PTB solution after rinse were immersed into 20 mL of 100 × 10^−6^ m ThT solution at room temperature for 15 min in the dark. After that, the lotus leaves were taken out and rinsed with sufficient water. The stained lotus leaves were dried and imaged by CLSM.^[^ ^35^ ^]^

### Pesticide Fixation Evaluation

A variety of pesticides were used in the experiment, and buprofezin (50%, SC) is used as an example for description. Buprofezin@water suspension and Buprofezin@BSA suspension liquid were formed by adding buprofezin (10 mg mL^−1^) in water and native BSA aqueous solution (10 mg mL^−1^), respectively. The Buprofezin@BSA was then divided into two parts, one of which was mixed with an equal volume of TCEP (50 × 10^−3^ m, pH = 4.5) to form Buprofezin@PTB. Five hundred microliters of the suspension was sprayed evenly onto plant leaf surfaces with a determined tilt angle of 30° from the ground at room temperature and incubated for 120 min at ambient conditions. Then, the amount of rinsing water was further increased from 6 mL to ≈2500 mm precipitation with a score force at 9.6 N through a self‐made device (Figure S12, Supporting Information). After air drying, 5 mL of 500 × 10^−3^ m VC solution was applied to the leaf surface for 30 min to disassemble the PTB nanofilm,^[^ ^60^ ^]^ VC solution was collected and extracted with 12 mL dichloromethane for four times to quantify the amount of pesticides retained on plant leaves. Ultraviolet–visible (UV–vis) spectroscopy was used to measure the absorption of buprofezin to determine the amount of buprofezin on the plant leaves.

### Weather Resistance Test

Hot air aging test: the PTB nanofilm coated on nano‐SiO_2_ superhydrophobic substrate was placed into oven at 80 °C for 30 days. Cold air aging test: the PTB nanofilm coated on nano‐SiO_2_ superhydrophobic substrate was placed into refrigerator at −24 °C for 30 days. Light aging test: the PTB nanofilm coated on nano‐SiO_2_ superhydrophobic substrate was placed under the sunlight lamp (power for 20 000 LX) for 30 days. pH 4 aging test: the PTB nanofilm coated on nano‐SiO_2_ superhydrophobic substrate was placed into water (pH 4) for 30 days. Microbial aging test: the PTB nanofilm coated on nano‐SiO_2_ superhydrophobic substrate was placed into the microorganism breeding environment at 37 °C for 90 days. The nano‐SiO_2_ based superhydrophobic surface was prepared by spin‐coating nano‐SiO_2_ superhydrophobic liquid on glass sheet and placing it into oven at 60 °C for 30 min.^[^ ^57^ ^]^

### Organic Solvents Resistance Test

The PTB nanofilm coated on lotus leaf surface was placed into ethanol, hexane, petroleum ether, chloroform, and DMF for 2 h.

### Control Efficacy on Snails

Forty snails were placed into four separate culture boxes with soil on the bottom, each with 10 snails at uniform size. Then 20 µL of pure water, PTB solution, metaldehyde suspension and the mixture of metaldehyde and PTB solution at a volume ratio of 1:1 were dropwise added to the exposed body of snail, respectively. The final concentration of metaldehyde is 100 mg mL^−1^. After 48 h, soaking them into water with the height of 1 cm to determine whether they are alive or dead. Finally, the number of the dead snails was counted, and the mortality of snails was calculated according to equation

(1) $$\mathrm{Mortality}\left(\%\right)=N_{t}/N_{0}\times 100\%$$

Where *N*_t_ is the number of dead snails at time of 48 h, and *N*_0_ is the total number of snails. All tests were carried out in triplicate.

### Farmland Experiment

The vineyard was divided into seven sections, with each section having an area of 50 m^2^ and three subdivisions in each section. The corn farmland was divided into seven sections, with each section having an area of 96 m^2^ and four subdivisions in each section. Each group in the experimental field was separated by a 5 m^2^ of open space. The corn and grape experimental fields are located in Xi 'an, China (108° 93′ E, 34° 17′ N). The experiment period was from June to September in 2020, and the weather temperature during this period was shown in Table S6 (Supporting Information). The density of the soil is 2.197 g mL^−1^, the pH of the soil is 6.64, and the composition and content of the soil is shown in Table S7 (Supporting Information).

### The Statistic of the Lesion Area

Ten grape leaves were randomly selected from each group of grape leaves and photographed. The yellow and brown lesions were in sharp contrast to the original green leaves and Image J software was used to analyze the lesion area on each leaf. The lesion area was calculated by the following expression

(2) $$\mathrm{Lesion}\;\;\mathrm{area}\;\;\left(\%\right)=S^{\prime }/S\times 100\%$$

where *S*’ is the area of lesion region, and *S* is the total area of each grape leaf.

### The Statistic of the Worm‐Eaten Ratio

And the worm‐eaten ratio of corn plants was recorded by the following method: the number of corn plants eaten by worms in each group was counted. The worm‐eaten ratio was calculated according to the expression

(3) $$\mathrm{Worm}-\mathrm{eaten}\;\;\mathrm{ratio}\;\;\left(\%\right)=N^{\prime }/N\times 100\%$$

where *N*’ is the number of corn plants eaten by worms, and *N* is the total number of corn plants.

## Conflict of Interest

The authors declare no conflict of interest.

## Author Contributions

H.S. and Y.L. contributed equally to this work. P.Y. and H.S. conceived and designed the project. P.Y., H.S., and Y.L. designed the experiments and performed all of the experiments with input from Y.G., C.F., C.L., and L.L.. P.Y., H.S., and Y.L. analyzed the data and prepared the figures. H.S. and P.Y. wrote the paper, with feedback from Y.L. and R.Q.

## Supporting information

Supporting Information

Supplemental Movie 1

Supplemental Movie 2

Supplemental Movie 3

Supplemental Movie 4

Supplemental Movie 5

Supplemental Movie 6

Supplemental Movie 7

Supplemental Movie 8

Supplemental Movie 9

## Data Availability

The data that support the findings of this study are available from the corresponding author upon reasonable request.
