# Supplementary material for: Amyloid‐Like Protein Aggregation Toward Pesticide Reduction
Source: Adv Sci (Weinh). 2022 Mar 8;9(13):2105106. doi: 10.1002/advs.202105106 (PMC9069373; doi:10.1002/advs.202105106)
Supplement: Supplementary file 1 — Supporting Information [file ADVS-9-2105106-s004.pdf]

ADVANCED SCIENCE
Open Access

# Supporting Information

for *Adv. Sci.*, DOI 10.1002/advs.202105106

Amyloid-Like Protein Aggregation Toward Pesticide Reduction

*Hao Su, Yongchun Liu, Yingtao Gao, Chengyu Fu, Chen Li, Rongrong Qin, Lei Liang and Peng Yang**

# Supporting Information

# Amyloid-Like Protein Aggregation towards Pesticide Reduction

*Hao Su, Yongchun Liu, Yingtao Gao, Chengyu Fu, Chen Li, Rongrong Qin, Lei Liang, Peng Yang**

H. Su, Dr. Y. Liu, Y. Gao, C. Fu, R. Qin, Prof. Dr. P. Yang

Key Laboratory of Applied Surface and Colloid Chemistry, Ministry

of Education, School of Chemistry and Chemical Engineering, Shaanxi Normal University, Xi'an 710119, China.

E-mail: yangpeng@snnu.edu.cn

Dr. C. Li, Dr. L. Liang

School of Chemistry and Chemical Engineering, Henan Institute of Science and Technology, Eastern HuaLan Avenue, Xinxiang, Henan 453003, China.

Supporting Information includes the following items:

Supporting Figures and Tables

Movie Files: Movies S1 to S9 (Web Enhanced Objects)

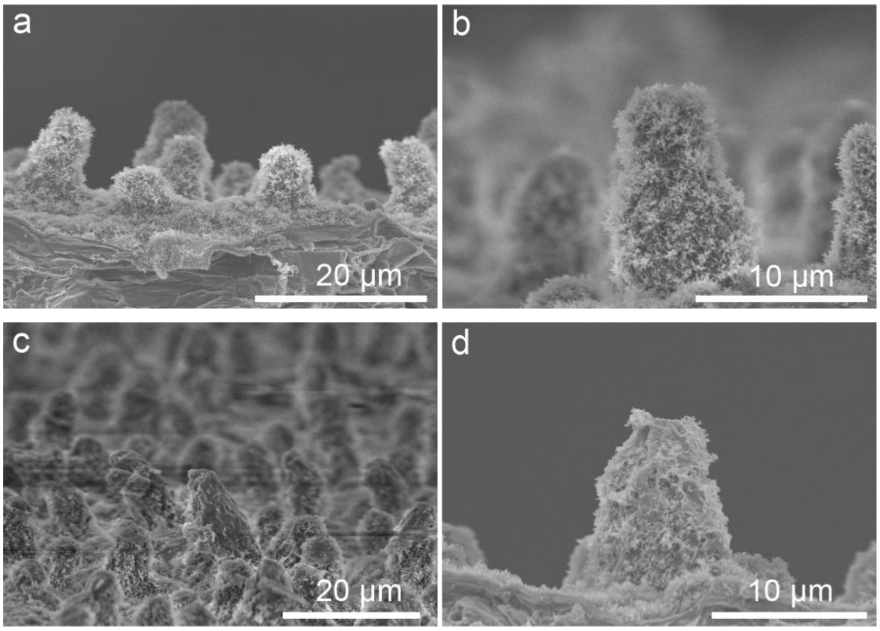


**Figure S1.** (a, b) The SEM images of the cross-section of the lotus leaf after spraying water and incubating for 120 min. (c, d) The SEM images of the cross-section of the lotus leaf after spraying native BSA solution and incubating for 120 min.

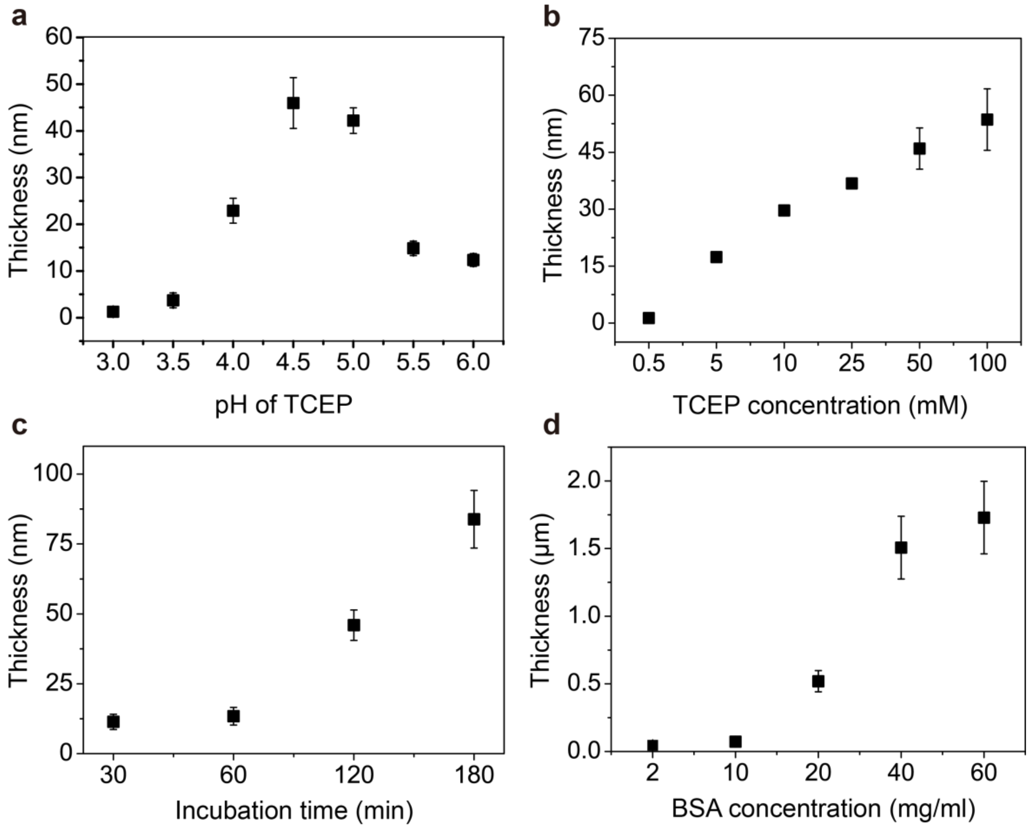


**Figure S2.** (a-d) The effect of the pH of TCEP solution (a), concentration of TCEP solution (b), incubation time (c) and BSA concentration (d) on the PTB nanofilm thickness coated on silicon wafer (Si). In (a), 2 mg/ml of BSA, 50 mM of TCEP solution and incubation for 120 min. In (b), 2 mg/ml of BSA, TCEP solution at pH 4.5 and incubation for 120 min. In (c), 2 mg/ml of BSA, 50 mM of TCEP solution at pH 4.5. In (d), 50 mM of TCEP solution at pH 4.5 and incubation for 120 min.

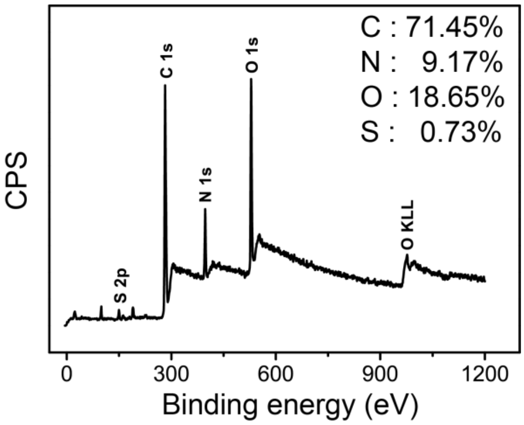


**Figure S3.** XPS survey characterization on the PTB nanofilm.

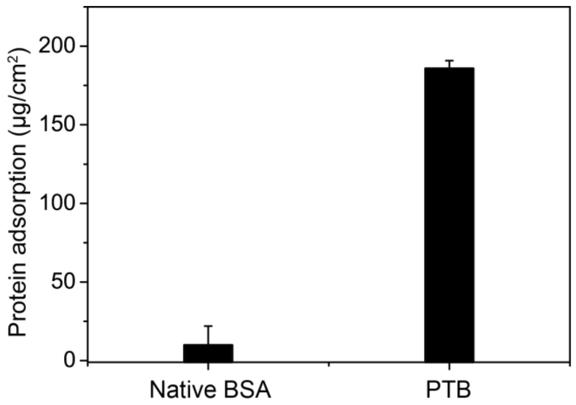


**Figure S4.** The bicinchoninic acid (BCA) protein assay for the adsorptions of native BSA and PTB on the lotus leaf.

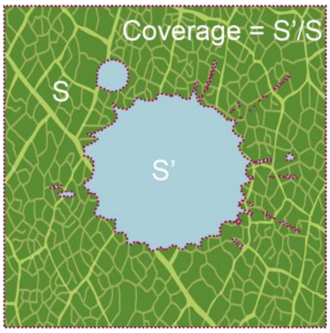

**Figure S5.** Schematic of surface coverage determination.

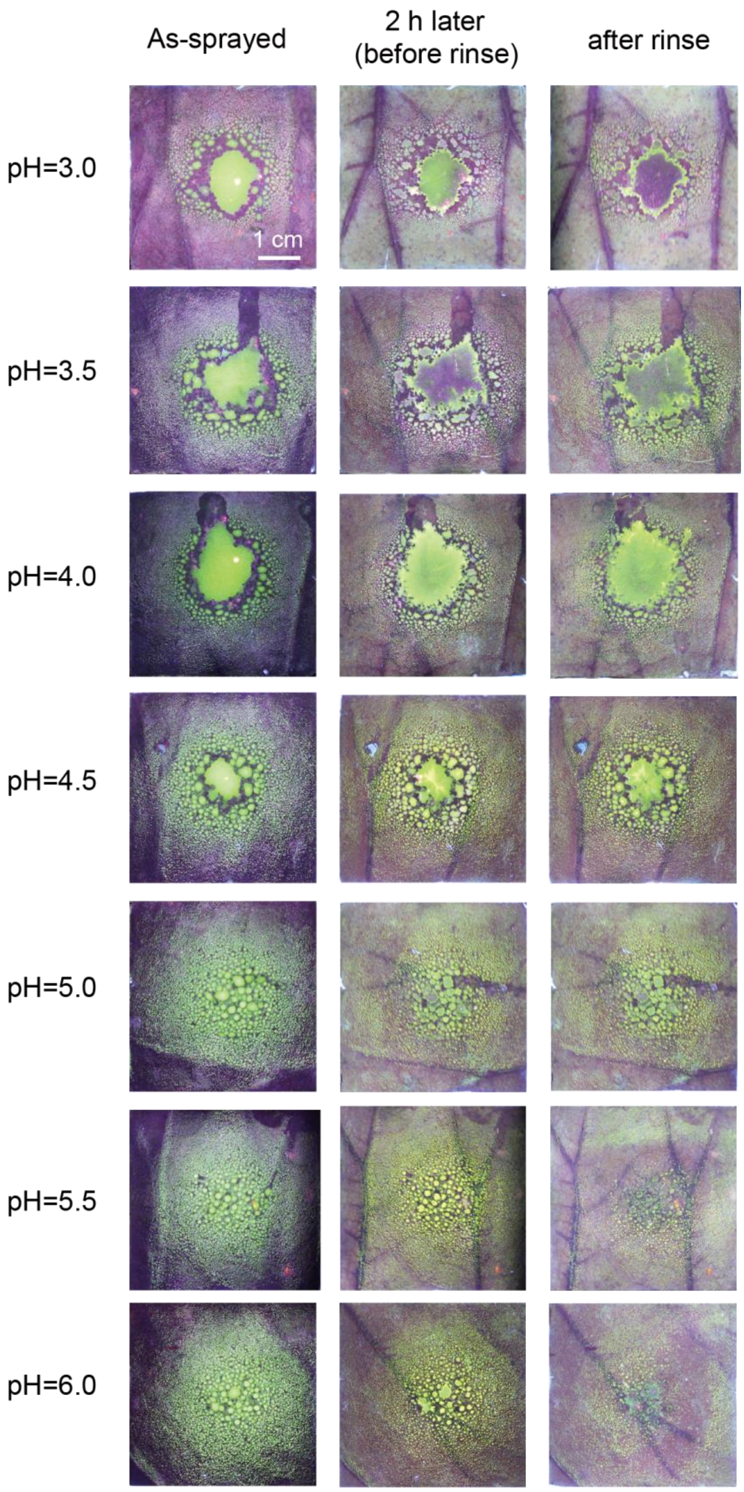


**Figure S6.** Fluorescent images of lotus leave surfaces (5×5 $cm^2$) after spraying 500 μl of the as-prepared PTB solution at different pH of TCEP solution. BSA-FITC was used for visualization.

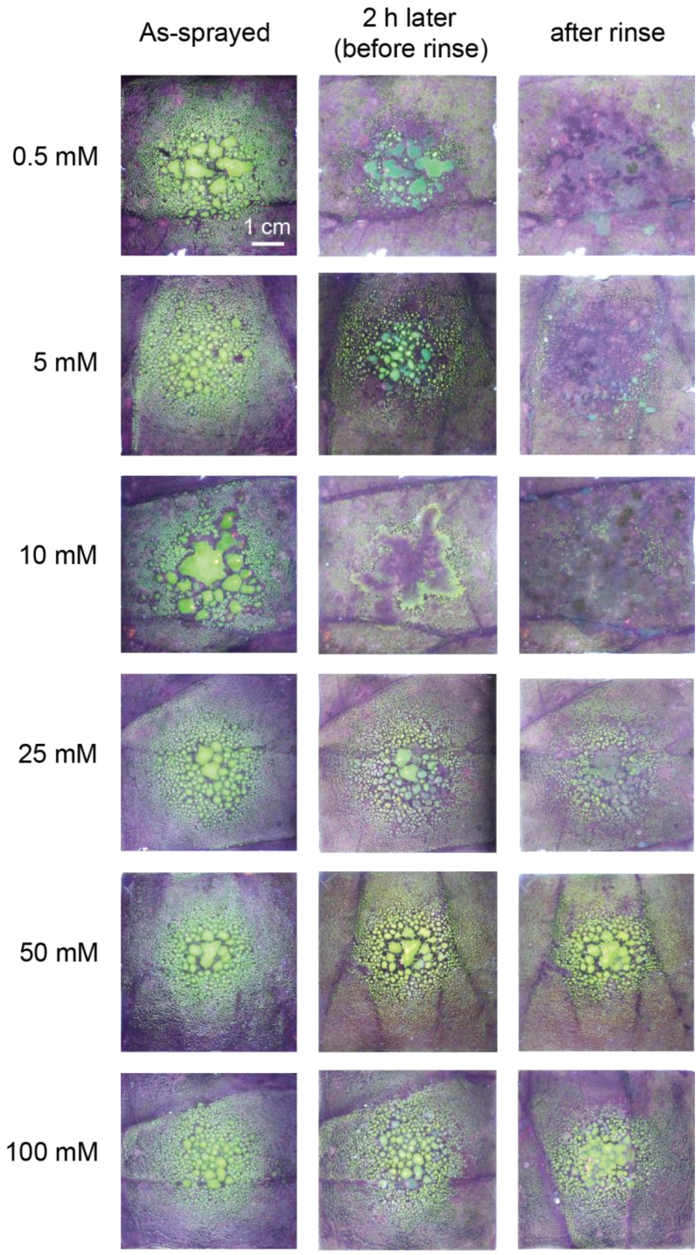


**Figure S7.** Fluorescent images of lotus leave surfaces (5×5 cm$^2$) after spraying 500 μl of the as-prepared PTB solution at different concentration of TCEP solution. BSA-FITC was used for visualization.

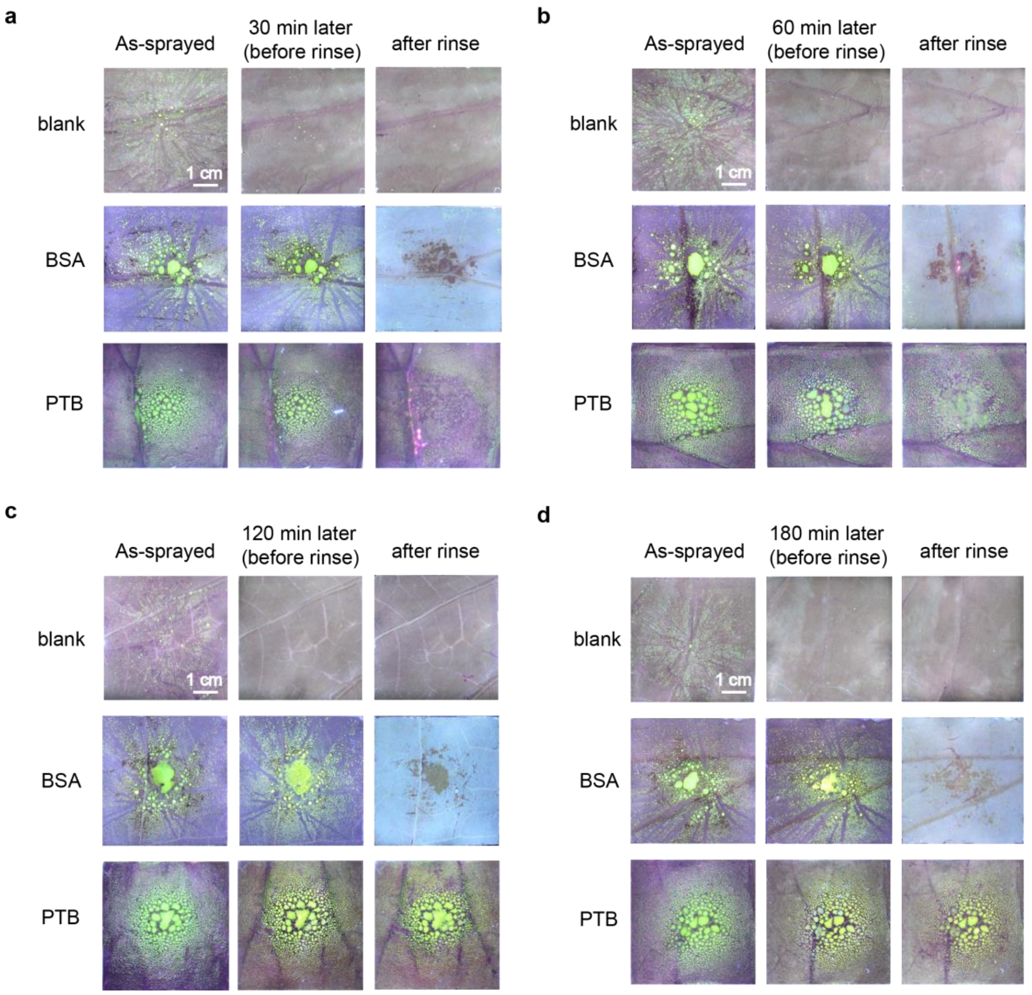


**Figure S8.** Fluorescent images of lotus leave surfaces (5×5 cm$^2$) after spraying 500 μl of water, native BSA solution or the as-prepared PTB solution and incubation at ambient condition for (a) 30 min, (b) 60 min, (c) 120 min and (d) 180 min. BSA-FITC was used for visualization.

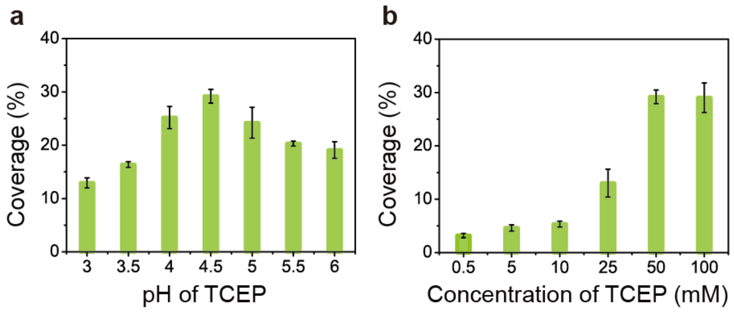


**Figure S9.** (a-b) Surface coverage of the lotus leaf surface by the PTB solution at different pH of TCEP solution (a), different concentration of TCEP solution (b). For these measurements, BSA concentration was 2 mg/ml.

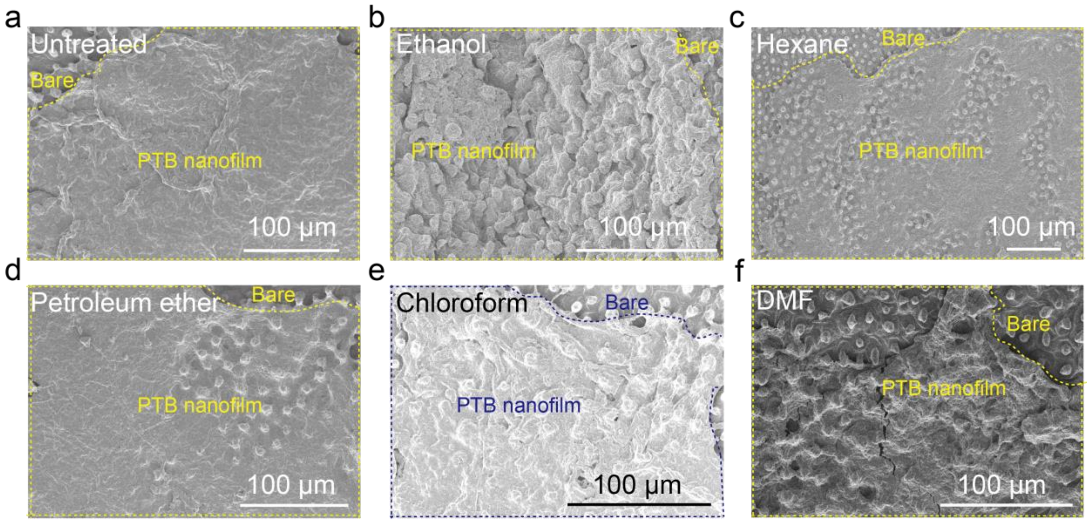


**Figure S10.** The stability tests of the PTB nanofilm after treatment in organic solvents. (a) SEM image of the lotus leaf covered by the PTB nanofilm. (b-f) SEM images of the PTB nanofilms on the lotus leave surfaces after treating by ethanol (b), hexane (c), petroleum ether (d), chloroform (e), and DMF (f) for 120 min. For clarity, the imaging is taken near the borderline between the PTB coating area and bare lotus leaf surface (as indicated by the dotted curve).

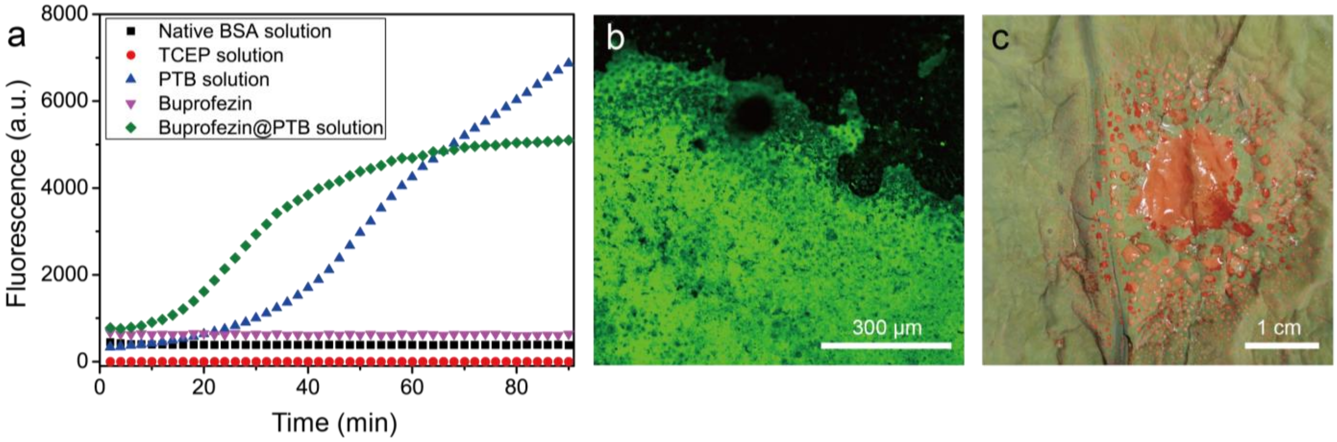


**Figure S11.** (a) The ThT fluorescence of buprofezin with different treatment**.** The ThT staining (b) and congo red staining (c) of the mixture of PTB nanofilm and buprofezin on lotus leaf surface.

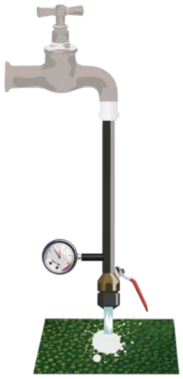

**Figure S12.** Schematic of a device that can measure the pressure of tap water to calculate the force applied to the lotus leaf surface. Inner diameter of tap water pipe is 0.8 cm. Adjust tap water pressure to 0.15 MPa, and the scour force (F) of water is calculated according to the equation: F (N) = P (Pa) ×S ($m^2$), where P is the pressure of water, and S is the cross-sectional area of inner pipe.

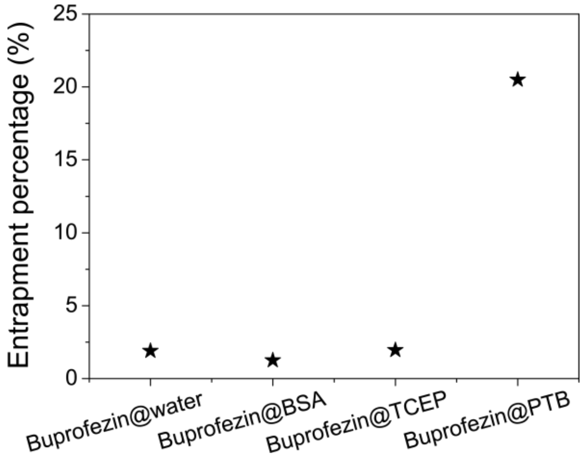


**Figure S13.** The entrapment percentage of buprofezin in water, native BSA, TCEP, and PTB groups on the lotus leaf.

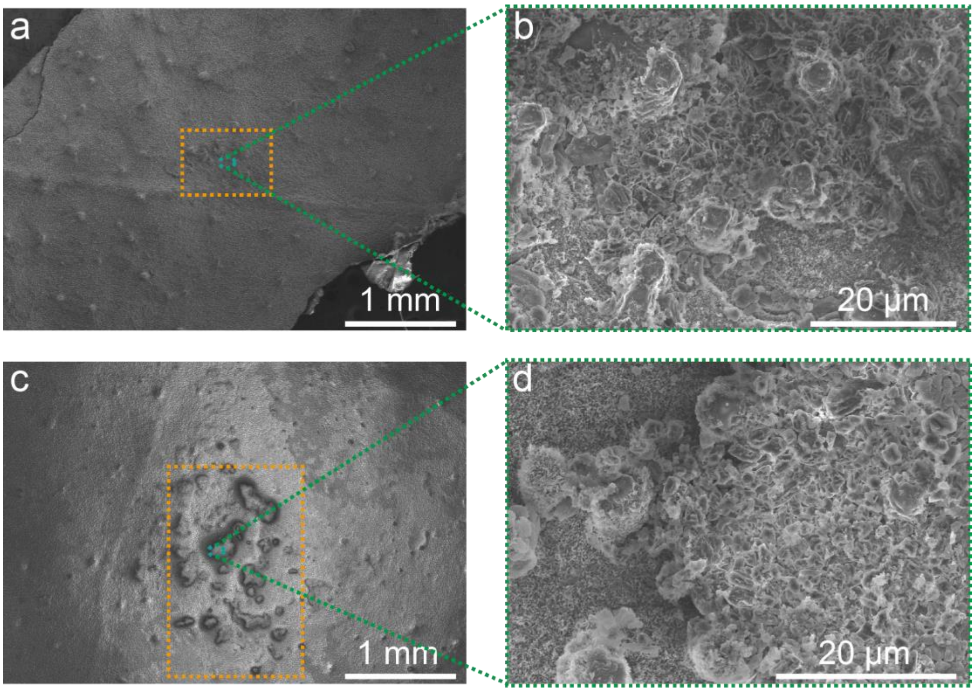


**Figure S14.** (a) SEM image of the lotus leaf surface with spraying the mixture of water and buprofezin (Buprofezin@water) after rinsing with water. The buprofezin retained on the lotus leaf was marked by an orange dotted rectangle. (b) The high magnification SEM image of buprofezin marked by a green dotted cycle in (a). (c) SEM image of the lotus leaf surface with spraying the mixture of native BSA and buprofezin (Buprofezin@BSA) after rinsing with water. The buprofezin retained on the lotus leaf was marked by an orange dotted rectangle. (d) The high magnification SEM image of buprofezin marked by a green dotted cycle in (c).

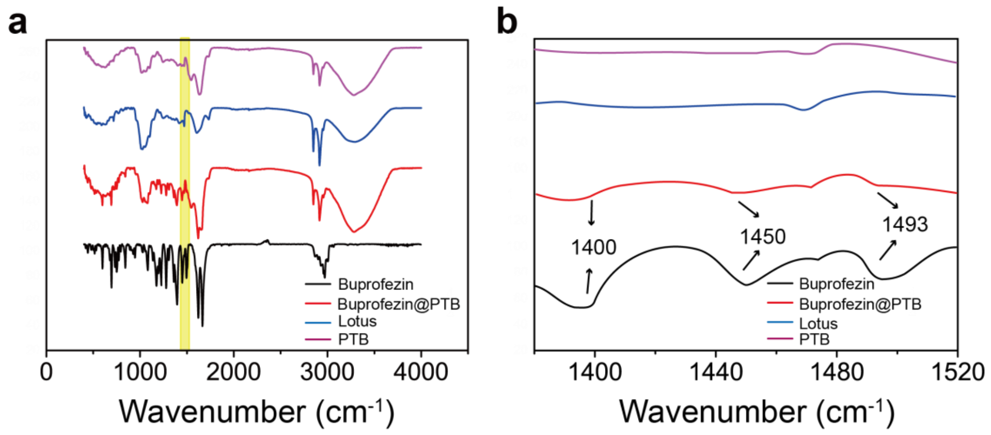


**Figure S15.** FTIR characterization on the PTB nanofilm. (a) The FTIR spectra of the lotus leaf, buprofezin, the PTB nanofilm and PTB nanofilm with buprofezin entrapped. (b) The skeleton vibration peaks of benzene ring in 1380~1520 cm$^{-1}$ for different groups in (a).

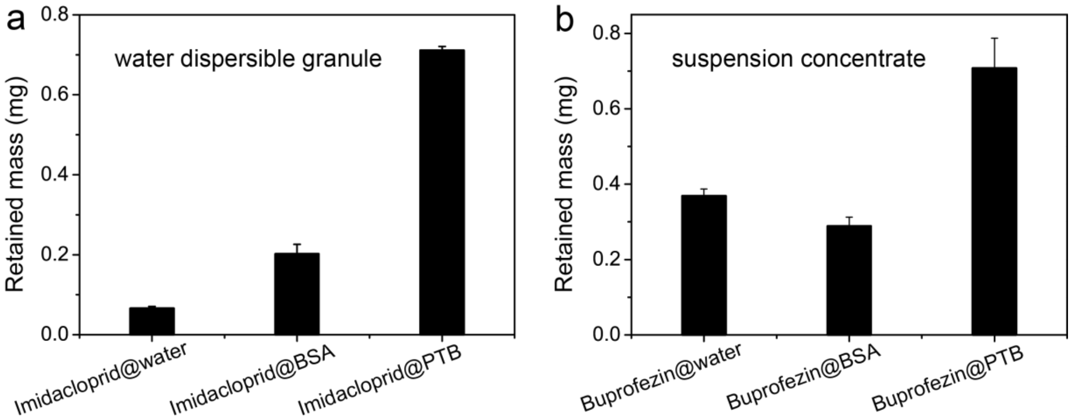


**Figure S16.** (a) The retained mass of imidacloprid (70%, water dispersible granule) on the lotus leaf after 6 ml water rinsing. (b) The retained mass of buprofezin (50%, suspension concentrate) on the lotus leaf after 6 ml water rinsing.

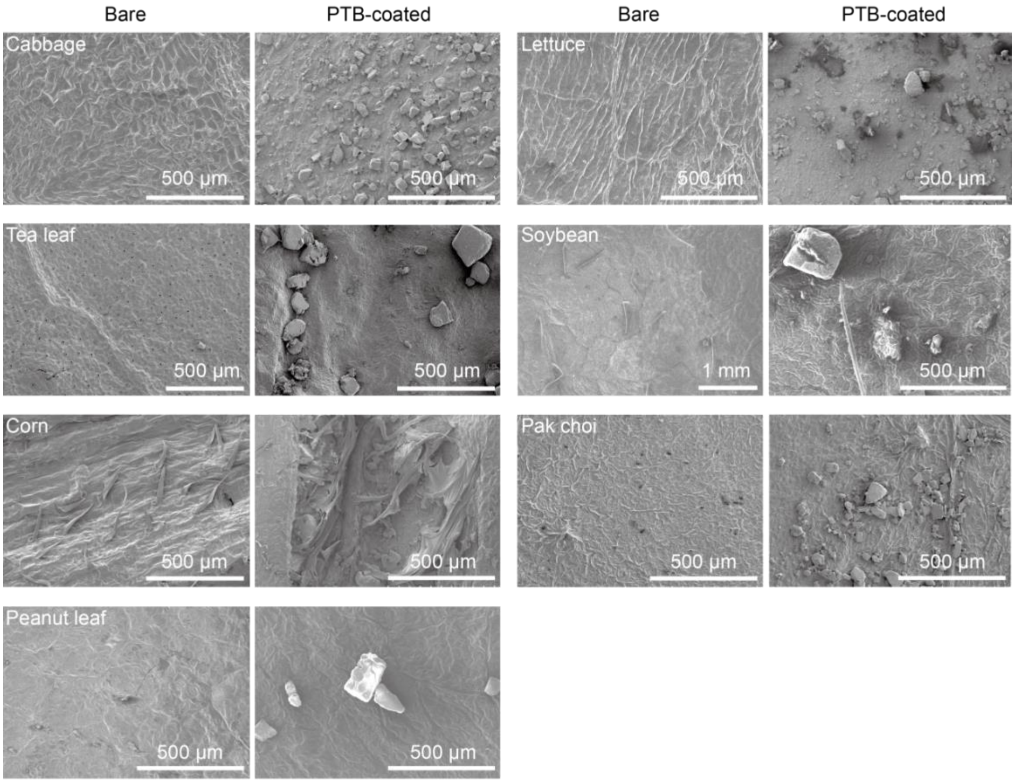


**Figure S17.** SEM images of different plant leave surfaces before (Bare) and after (PTB-coated) spraying the mixture of PTB suspension and buprofezin (95% in water) and rinsing with water.

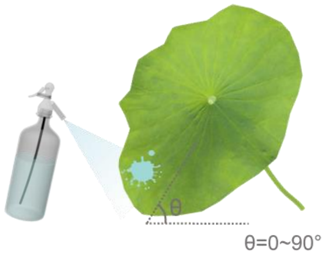


**Figure S18.** Schematic of spraying the mixture of pesticide suspension on lotus leaves at incidental angles of 0, 30, 60 and 90 °.

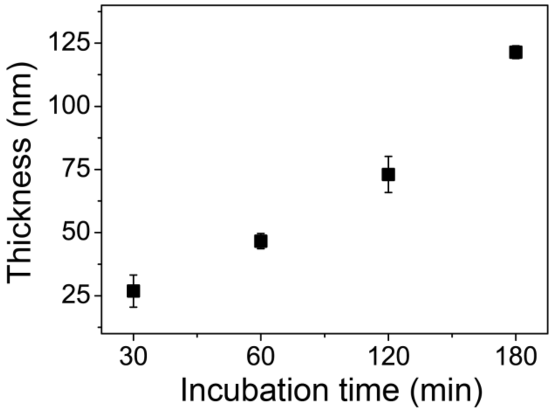


**Figure S19.** The effect of the incubation time on the PTB nanofilm thickness coated on silicon wafer (Si). The PTB nanofilm was prepared by mixing 10 mg/ml of BSA with 50 mM of TCEP (pH 4.5) at the volume ratio of 1:1.

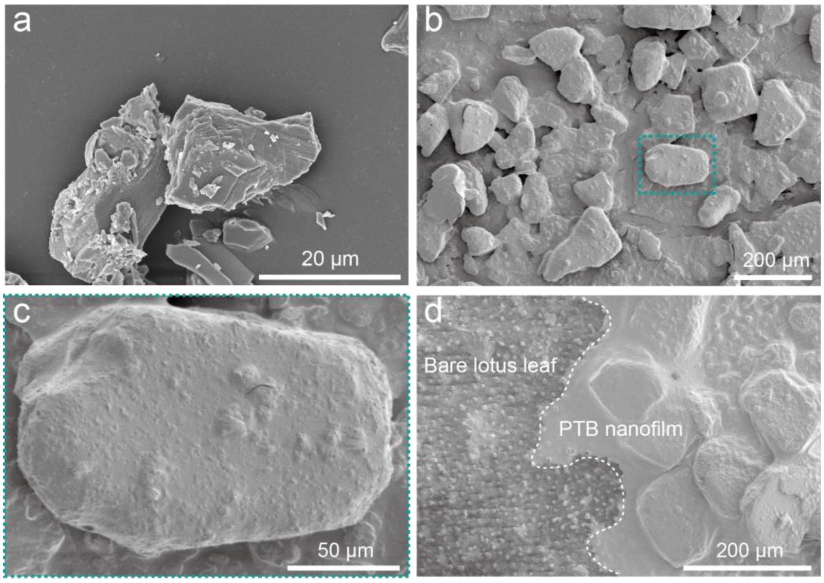


**Figure S20.** The relationship between the protein membrane and pesticide. (a) SEM image of bare buprofezin (95%, technical material) particles. (b) SEM image of buprofezin particles coated by the PTB nanofilm. (c) The high magnification SEM image of buprofezin marked by a rectangle in (b). (d) SEM image of buprofezin particles covered by the PTB nanofilm. For clarity, the image is taken near the borderline between the PTB coating area and bare lotus leaf surface (as indicated by a white dotted curve).

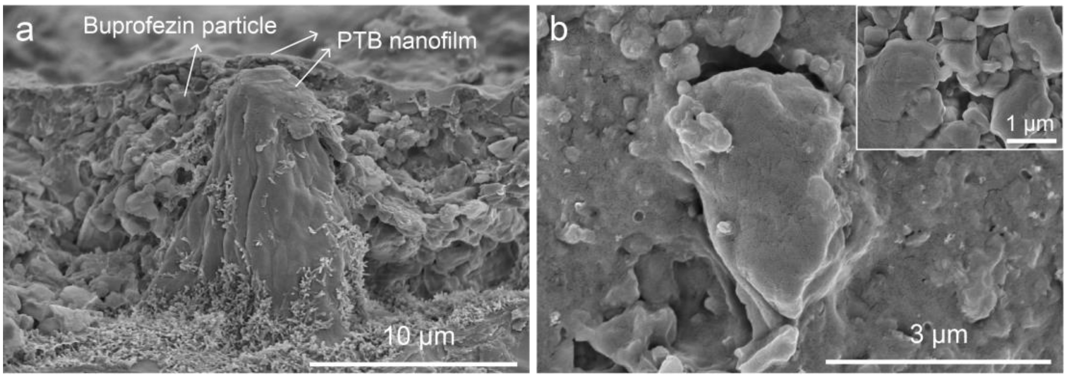


**Figure S21.** (a) The SEM image of the cross-section of the PTB nanofilm entrapping buprofezin (50%, SC) on the lotus leaf micropillar. (b) The SEM image of buprofezin particles coated with PTB nanofilm, the SEM image in the inset showing the bare buprofezin particles.

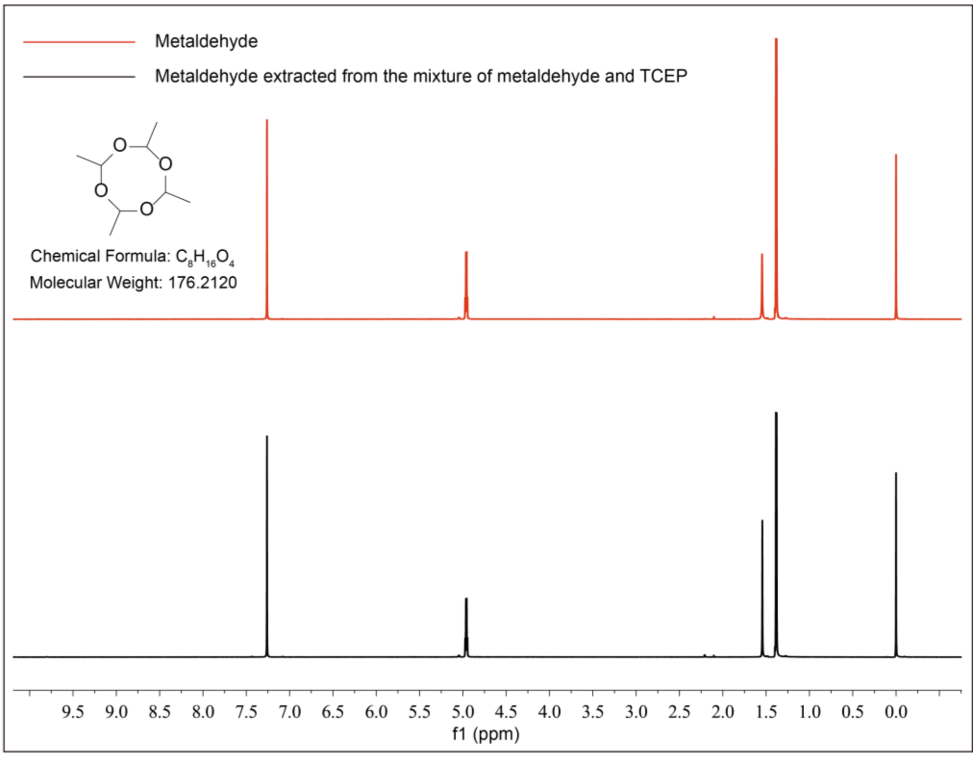


**Figure S22.** $^1$H Nuclear Magnetic Resonance (NMR) spectra of pure metaldehyde (red line) and metaldehyde extracted from the mixture of metaldehyde and TCEP (black line), showing the presence of metaldehyde (d: 1.38 ppm, q: 5.2 ppm).

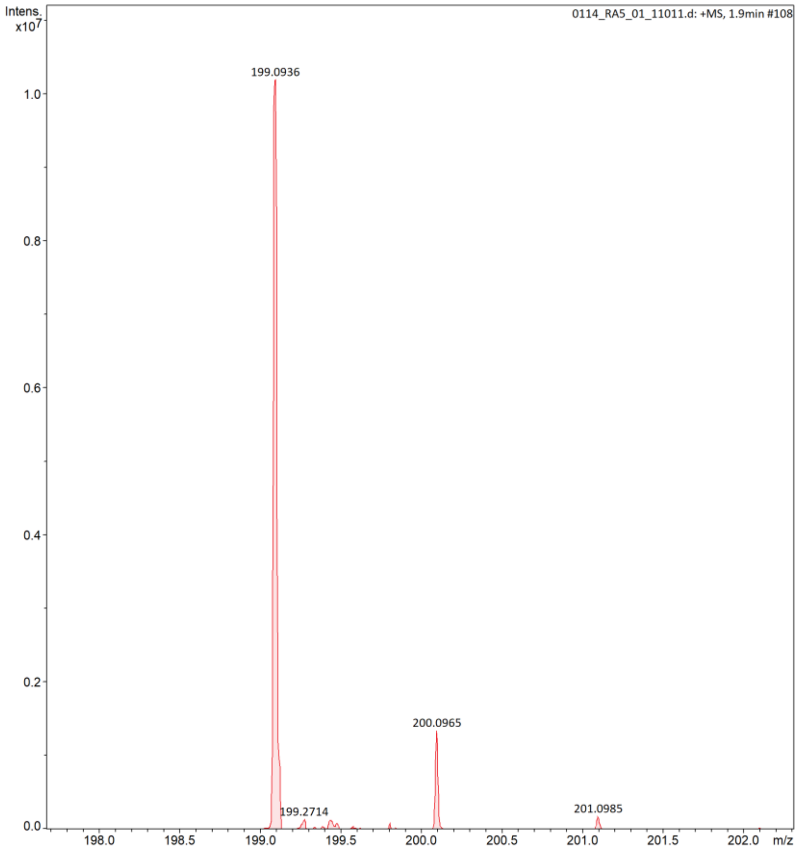


**Figure S23.** Electrospray Ionization Mass Spectrometry (ESI-MS) characterization of pure metaldehyde and metaldehyde extracted from the mixture of metaldehyde and TCEP. ESI-MS: calculated value for pure metaldehyde (C8H16O4$Na^+$ $[M+Na]^+$): 199.0941; measured value for metaldehyde extracted from the mixture of metaldehyde and TCEP: 199.0936.

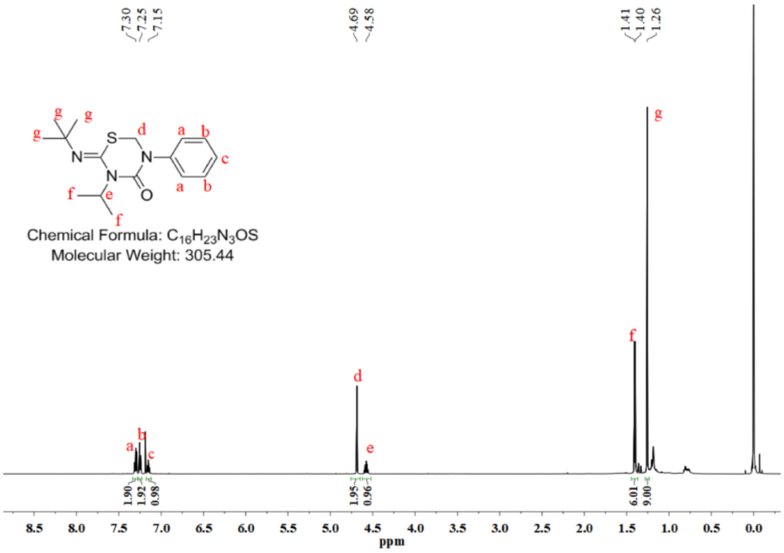


**Figure S24.** $^{1}H$ NMR characterization of buprofezin fixed by the PTB nanofilm.

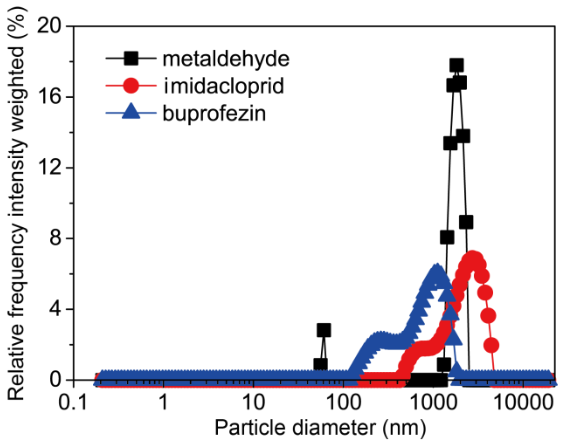


**Figure S25.** The size distribution of metaldehyde (80%, wettable powder), imidacloprid (70%, water dispersible granule) and buprofezin (50%, suspension concentrate) in water.

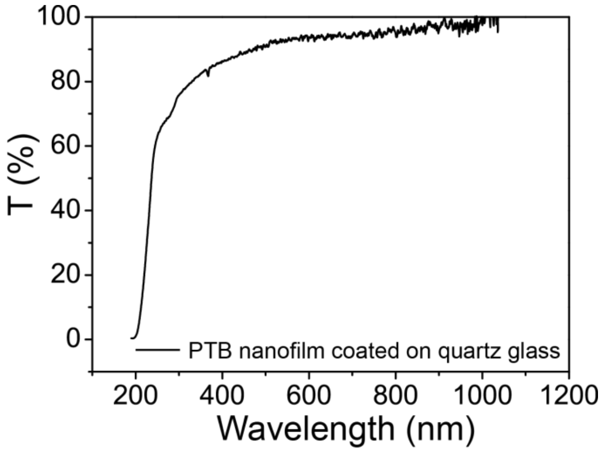


**Figure S26.** Transmittance of the PTB nanofilm coated on quartz glass.

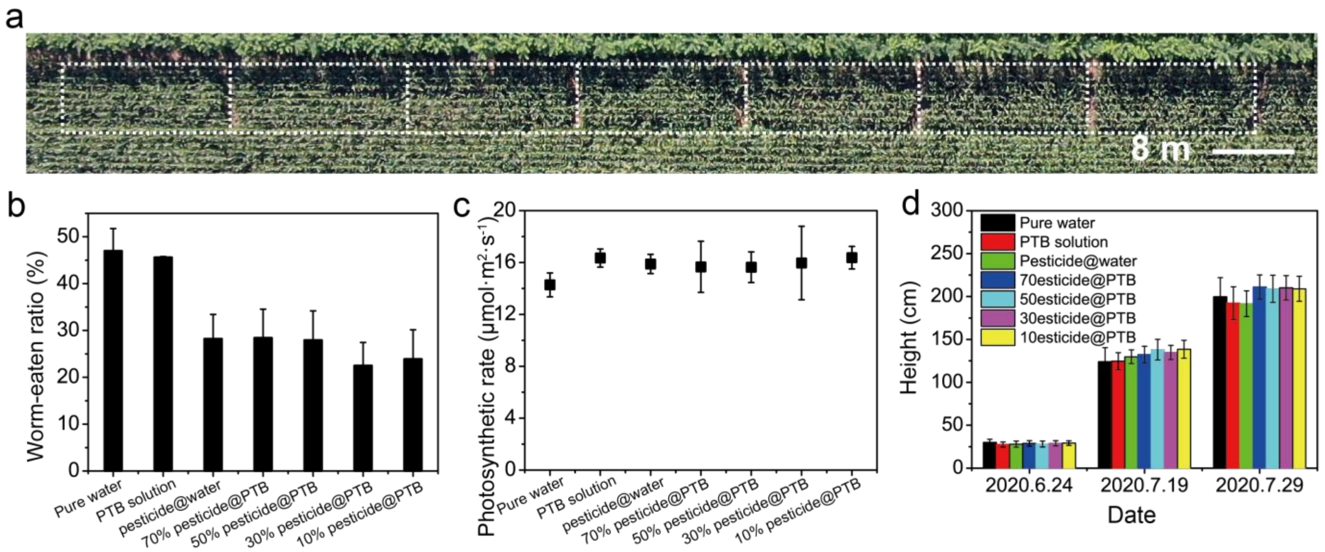


**Figure S27.** (a) The aerial photo, (b)worm-eaten ratio, (c) photosynthetic rate and (d) height of corns in corn field sections corresponding to the groups of pure water, PTB solution, the mixture of pesticide and water (pesticide@water), the mixture of 70% pesticide and PTB (70% pesticide@PTB), the mixture of 50% pesticide and PTB (50% pesticide@PTB), the mixture of 30% pesticide and PTB (30% pesticide@PTB), and the mixture of 10% pesticide and PTB (10% pesticide@PTB).

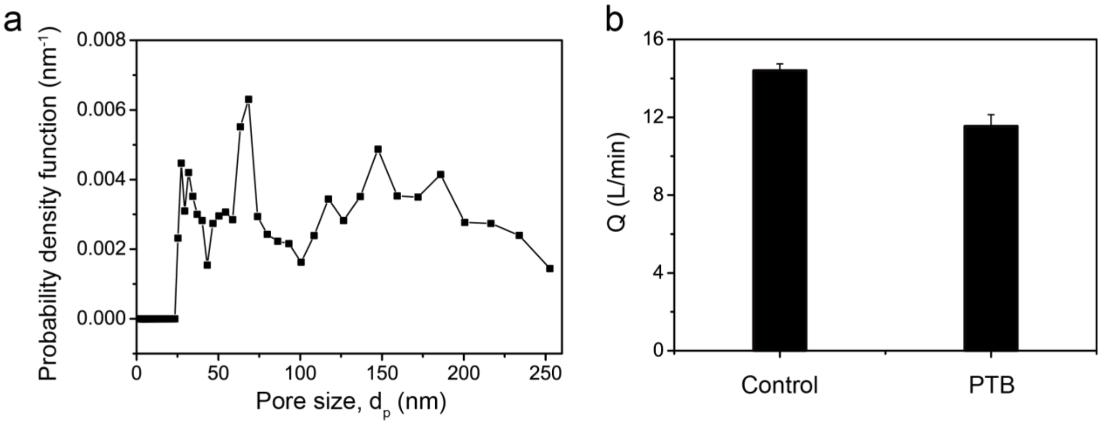


**Figure S28.** (a) The pore size distribution of the PTB nanofilm. The PTB nanofilm was prepared with 2 mg/ml BSA, 50 mM TCEP (pH 4.5) and incubation for 2 hours. (b) The air permeability of nylon filter cloth with a pore size of 15 μm (control sample), and nylon filter cloth covered by the PTB nanofilm (PTB sample).

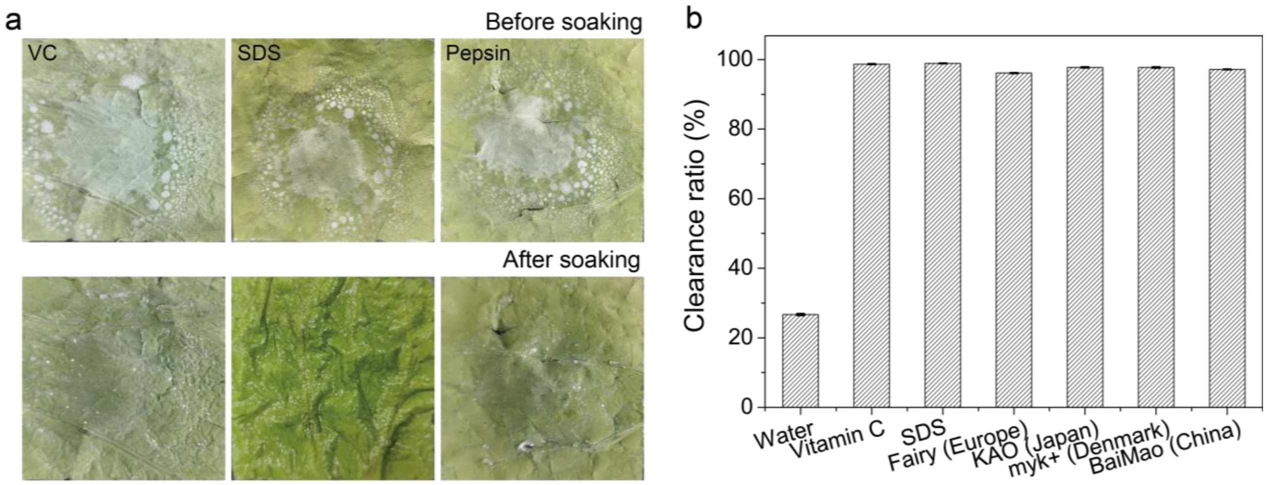


**Figure S29.** (a) The photograph of PTB nanofilm on the lotus leaf before and after soaking by VC (0.5 M), SDS (1%), and pepsin solution (0.5%). (b) The clearance ratio of pesticides on lotus leaves by VC, SDS and other detergent solution.

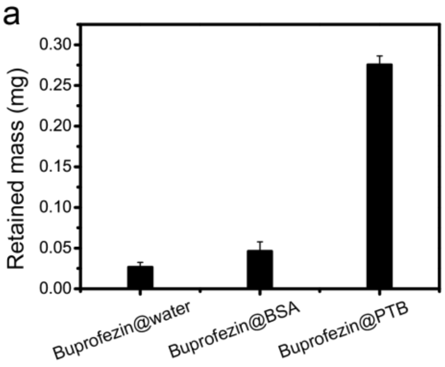


b

| Reagent | Cost of normal formulation (dollars per hectare) | Cost of optimized formulation (dollars per hectare) |
|---|---|---|
| BSA | —— | 45 |
| TCEP | —— | 20 |
| The pesticides used in the article | 87-121 | 8.7-12.1 |
| Total price | 87-121 | 73-77 |

**Figure S30.** (a) Retained mass of commercial buprofezin in water (Buprofezin@water group), in native BSA solution (Buprofezin@BSA group), in the as-prepared PTB solution with BSA concentration being 1 mg/ml, and TCEP concentration being 5 mM (Buprofezin@PTB group) on the lotus leaves after water washing with a scour force at 9.6 N and a rainfall precipitation at 2500 mm. (b) The cost per hectare for commercial pesticide used in our farmland experiment and commercial pesticide used in our experiment with the PTB formulation.

**Table S1.** Formulation of commercial buprofezin (50%, SC).

| Pesticide formula ingredients | Percentage composition (%) |
|---|---|
| buprofezin | 50 |
| dispersing agent (SD-06) | 2 |
| wetting agent (SR-08) | 1 |
| glycol | 4 |
| Xanthan gum | 2 |
| magnesium aluminium silicate | 0.5 |
| sodium benzoate | 0.2 |
| organosilicon defoamer (AFE-3168) | 0.2 |
| deionized water | 40.1 |

**Table S2.** Pesticide function and their use area.

| Classification | Pesticides | Function | Whether to allow use | Use area |
|---|---|---|---|---|
| Insecticide | Buprofezin | For controlling pests of stinging mouthparts | Yes | More than 80 countries and regions |
| | Imidacloprid | Controlling Cicadaceae and Planthoppers on rice | Yes | Asia, Africa, USA and South America |
| Fungicide | Diethofencarb | For the control of gray mold of vegetables and fruits | Yes | More than 80 countries and regions |
| Acaricide | Tetrapyrazine | For controlling mites on the canopy of apples and other fruit trees | Yes | More than 80 countries and regions |
| Herbicide | Pendimethalin | Used to control annual grasses and certain broad-leaved weeds | Yes | More than 80 countries and regions |

**Table S3.** Pesticides used in the vineyard.

| Classification | Commercial pesticides | Pesticide formulations | Whether to allow use |
|---|---|---|---|
| Fungicide | Dimethomorph ·cream urea cyanide | 70%, water dispersible granule | Yes |
| Fungicide | Boscalid ·Iprodione | 35%, suspending agent | Yes |
| Fungicide | Penconazole | 20%, emulsion in water | Yes |
| Insecticide | Thiamethoxam ·Lambda-cyhalothrin | 17%, suspending agent | Yes |
| Insecticide | Methylamabamectin benzoate ·mites nitrile | 12%, suspending agent | Yes |
| Fungicide | Pentazole ·cytosyl ester | 40%, suspending agent | Yes |

**Table S4.** Pesticides used in the corn farmland.

| Classification | Commercial pesticides | Pesticide formulations | Whether to allow use |
|---|---|---|---|
| Insecticide | Lufenuron·chlorfenapyr | 12%, suspending agent | Yes |
| Fungicide | Thiophanate-methyl | 50%, suspending agent | Yes |
| Insecticide | Imidacloprid | 70%, water dispersible granule | Yes |
| Insecticide | Chlorantraniliprole | 20%, suspending agent | Yes |

**Table S5.** Weather conditions during the experiment period in a real farmland.

| Date | 24-hour temperature (K) | Weather | Rainfall (mm) |
|---|---|---|---|
| 2020.9.3 | 289.15~303.15 | Sunny | 0 |
| 2020.9.4 | 290.15~302.15 | Partly cloudy | 0 |
| 2020.9.5 | 293.15~302.15 | Light rain | 8.7 |
| 2020.9.6 | 290.15~303.15 | Sunny | 0 |
| 2020.9.7 | 290.15~305.15 | Sunny | 0 |
| 2020.9.8 | 292.15~305.15 | Sunny to cloudy | 0 |
| 2020.9.9 | 290.15~295.15 | Light rain | 7.4 |
| 2020.9.10 | 290.15~301.15 | Partly cloudy | 0 |
| 2020.9.11 | 291.15~301.15 | Sunny to cloudy | 0 |
| 2020.9.12 | 290.15~301.15 | Cloudy to overcast | 0 |

**Table S6.** Weather temperature during the farmland experiment period.

| Date | The average high temperature (K) | The average low temperature (K) |
|---|---|---|
| 2020.06 | 301.15 | 292.15 |
| 2020.07 | 301.15 | 293.15 |
| 2020.08 | 300.15 | 293.15 |
| 2020.09 | 298.15 | 289.15 |

**Table S7.** The composition and content of the soil.

| Composition | Content (wt%) |
|---|---|
| silicate | 37-38 |
| calcium carbonate | 12-13 |
| silicon dioxide | 18-19 |
| humus | 9-10 |
| water | 22-23 |
